# Stressing out—carp edema virus induces stress and modulates immune response in common carp

**DOI:** 10.3389/fimmu.2024.1350197

**Published:** 2024-03-21

**Authors:** Maria Zawisza, Alexander Rebl, Felix Teitge, Barbara Krzystyniak, Veronika Piackova, David Gela, Martin Kocour, Magdalena Chadzinska, Mikolaj Adamek, Krzysztof Rakus

**Affiliations:** ^1^ Department of Evolutionary Immunology, Institute of Zoology and Biomedical Sciences, Faculty of Biology, Jagiellonian University, Krakow, Poland; ^2^ Doctoral School of Exact and Natural Sciences, Jagiellonian University, Krakow, Poland; ^3^ Research Institute for Farm Animal Biology (FBN), Dummerstorf, Germany; ^4^ Fish Disease Research Unit, Institute for Parasitology, University of Veterinary Medicine Hannover, Hannover, Germany; ^5^ University of South Bohemia in Ceske Budejovice, Faculty of Fisheries and Protection of Waters, South Bohemian Research Centre of Aquaculture and Biodiversity of Hydrocenoses, Vodnany, Czechia

**Keywords:** fish poxviruses, carp edema virus, CEV, koi sleepy disease, stress, immunomodulation, common carp

## Abstract

**Introduction:**

Carp edema virus (CEV) is a fish poxvirus that primarily infects the gills of common carp. CEV causes koi sleepy disease (KSD), which is highly contagious and can result in mortality of up to 100%.

**Methods:**

In the present study, we analyzed the stress and immune responses during KSD in two strains of common carp with different resistance to CEV: susceptible koi and resistant Amur sazan. Experiments were performed at two temperatures: 12°C and 18°C. In the case of koi carp, we also analyzed the effect of supplementation of 0.6% NaCl into tank water, which prevents mortality of the CEV-infected fish (salt rescue model).

**Results:**

We found that CEV-infected koi kept at 18°C had the highest viral load, which correlated with the most severe histopathological changes in the gills. CEV infection resulted in the activation of stress response reflected by the upregulated expression of genes involved in stress response in the stress axis organs and increased levels of cortisol and glucose in the blood plasma. These changes were the most pronounced in CEV-infected koi kept at 18°C. At both temperatures, the activation of antiviral immune response was observed in koi kept under freshwater and NaCl conditions upon CEV infection. Interestingly, a clear downregulation of the expression of adaptive immune genes was observed in CEV-infected koi kept under freshwater at 18°C.

**Conclusion:**

CEV induces a stress response and modulates adaptive immune response in koi, and this is correlated with the level of viral load and disease development.

## 1 Introduction

Gill diseases seriously affect fish health and have a very negative impact on aquaculture mainly because of the multifunctional properties of the gills in fish physiology (1–3). Carp edema virus (CEV), a fish-infecting poxvirus, infects predominantly the gills of common carp and its ornamental koi varieties. CEV infection resulted in severe gill disease known as koi sleepy disease (KSD), which is fatal for the majority of infected fish (4, 5). KSD has been known since 1974, when it emerged in Japanese koi populations (6). However, CEV was detected in common carp and koi populations in many countries in Asia, Europe, and North America (6–11). There are currently three known distinct genogroups of CEV with a different geographical distribution. Genogroup I was found mostly in common carp in Europe and in the USA, while genogroup IIa contains viral isolates predominantly from diseased koi carp around the world. The third genogroup IIb has been detected in both common carp and koi, mainly in Europe, and phylogenetic analyses reveals that it belongs between the two previous genogroups (4, 9, 11).

One of the characteristic clinical signs of the CEV infection is coma-like behavior in affected fish, which manifests itself by the specimen lying on one side of the body and by poor response to stimuli. Other clinical signs include enopthalmia (sunken eyes), swelling and necrotic gills, skin hemorrhages and lesions, and white areas on the skin caused by local hyperproduction of mucus (12, 13). The observed histopathological changes include hyperplasia and hypertrophy of branchial epithelial cells, as well as occlusion of the branchial intralamellar spaces, and fusion of secondary lamellae are observed (5, 6, 14). Infiltration of inflammatory cells in the gills is also present (5, 14). CEV infection causes disturbances of the respiratory function, acid–base regulation, osmoregulatory function or nitrogenous waste excretion from the gills, resulting together in hypoxia, hyponatremia, and hyperammonemia (15).

Interestingly, high mortality of fish due to CEV infection can be prevented by salt treatment (6). A 0.6% NaCl solution (w/v) in tanks allowed fish to restore sodium concentration and balance ammonia level and prevented mortality of CEV-infected carp (14). Furthermore, water temperature is considered a key factor influencing the spread of the virus, and natural outbreaks and mortality during CEV infection usually occur when water temperature is between 12°C and 25°C (6, 16).

Due to its severe effect on the function of the gills, CEV infection might cause physiological distress in infected fish, which can affect many biological functions, including the immune response. In teleosts, the stress response involves activation of the hypothalamo-pituitary-interrenal (HPI) axis, which is analogous to the hypothalamic–pituitary–adrenal (HPA) axis of higher vertebrates (17). In general, when stress signals are perceived, the neurons of the hypothalamic nucleus preopticus (NPO) produce and secrete, among others, the corticotropin-releasing hormone (CRH). CRH interacts with its receptor (CRH-R1) on the corticotrope cells of the pituitary gland (PIT) and stimulates the release of adrenocorticotropic hormone (ACTH), which is cleaved from its precursor proopiomelanocortin (POMC). ACTH then stimulates interrenal cells of the head kidney (HK) and induces the transfer of cholesterol to the inner mitochondrial membrane and its conversion to cortisol (18). During the stress response, plasma cortisol levels can rapidly rise. Inside the cell, cortisol binds to intracellular glucocorticoid receptors (GRs), which then translocate to the nucleus to activate or repress transcription of effector genes *via* an interaction with glucocorticoid response elements (GRE) (19, 20). In addition, GRs can interact with other transcription factors, such as nuclear factor kappa B (NF-κB) and activator protein (AP-1), and, therefore, regulate expression of cytokines and their receptors (21). In both mammals and in fish, GRs have been described in immune cells (22). Interestingly, teleost fish have three different nuclear glucocorticoid receptors as a result of gene duplication (GR1 and GR2) and alternative splicing (GR1a and GR1b). These receptors possess differential sensitivity to cortisol and require basal (GR2) or stress (GR1) levels of cortisol (23, 24). In teleosts, cortisol can also be bound by the mineralocorticoid receptor (MR) and be involved in osmoregulation by inducing Na^+^/K^+^-ATPase (NKA) activity in the gills (25).

Cortisol induces immunosuppression, e.g., by altering leukocyte redistribution, production of antibodies and activating NK cells (26). Moreover, during stress response, the induced expression of genes encoding the pro-inflammatory cytokine IL-1β and its receptor IL-1bR was noted in selected organs of the HPI axis (27, 28). IL-1β is one of the key pro-inflammatory cytokines involved in brain signaling during stress leading to the activation of the stress axis (17, 29, 30).

Different strains of common carp show high (Amur sazan—wild carp) or low (koi carp) resistance to CEV (5, 14). Koi carp develop clinical signs around 6 to 9 days post exposure with mortality reaching up to 100%, whereas Amur sazan do not show any clinical signs or mortality. Other carp lines studied under laboratory conditions (Ropsha carp and Prerov carp) also did not develop any clinical signs and did not show mortality during CEV infection. Moreover, susceptible koi carp has significantly higher viral load in the gills compared to resistant Amur, Ropsha, and Prerov carp (5). Interestingly, the previous study focusing on type I interferon (IFN) response did not explain the differences in the resistance to CEV between carp strains (5). Importantly, CEV infection resulted in leukopenia and significant downregulation of the expression of *cd4*, *tcra2*, and *igm* genes in the gills of susceptible koi carp suggesting that the virus has an immunomodulatory effect (14). However, the decrease in the number of leukocytes in blood does not automatically equal suppression, as it may also mean redistribution of the cells. Nevertheless, studies on the effects of stress and a deeper analysis of the immune response have not yet been carried out in relation to CEV infection.

The main aim of our work was to study the impact of stress and immune responses on the outcome of KSD in Amur and koi carp strains, which show different levels of resistance to CEV. We also analyzed the effect of different water temperatures (12°C and 18°C) on the course of CEV infection. Our results clearly demonstrate that viral replication is higher at 18°C than at 12°C and that CEV activates stress response in infected koi carp. Furthermore, downregulation of genes involved in the adaptive immune response was observed in CEV-infected koi.

## 2 Materials and methods

### 2.1 Fish

Naïve common carp fry of Amur wild carp (Amur sazan, AS) and koi strains were obtained from the University of South Bohemia in Ceske Budejovice, Faculty of Fisheries and Protection of Waters, located in Vodnany, Czech Republic. Fish were raised in a recirculation system filled with tap water at 20°C and fed a commercial feed (Perla Plus, Skretting Norway) at a rate of 1% body weight per day. Prior to their use in infection experiments, fish had been confirmed to be free of ectoparasites by means of fresh skin and gill surface smears and examination using light microscopy. They were also confirmed to be free of specific viruses by means of RT-qPCR assays as described earlier (5, 31). These assays included the following viruses that infect common carp: CEV, cyprinid herpesvirus 3 (CyHV-3), spring viremia of carp virus (SVCV), and common carp paramyxovirus (CCPV).

### 2.2 Ethics

All experiments were carried out in accordance with national and international regulations for experimentation with animals, under approval of the 2nd Local Ethics Committee in Krakow, Poland (no. 355/2021).

### 2.3 Stress model

Amur sazan and koi carp (approximately 150 g) were kept at the same tank at 20°C for 3 weeks. Prolonged restraint stress (24 h) was induced by immobilizing fish in nets as described previously (32). Briefly, fish (n = 6 from each strain) were stressed by restraining each fish in a net and placing them in the plastic tank (120 L) with aeration at 20°C. Fish restrained in the net were in full contact with the water for 24 h. During the stress challenge, fish were not fed. After 24 h of the restraint stress, fish were immediately transferred all at once to a tank with an overdose of a buffered solution of MS-222 in water (0.4 g/L) and were euthanized prior to blood and tissues collection. Control fish were sampled following rapid netting and euthanized prior to blood and tissues collection immediately before sampling of fish from the stress group.

### 2.4 CEV infection model

Amur sazan and koi carp (approximately 150 g) were infected with CEV by cohabitation with clinically affected, virus-shedding donor fish, since propagation of this virus *in vitro* has not been established yet (12). Infection was performed with CEV from genogroup IIa at two temperatures: 12°C and 18°C. Prior to infection, fish were divided into six groups (n = 10 fish in each group): (i) control koi (Ctr Koi), (ii) CEV-infected koi (CEV Koi), (iii) salt-treated control koi (Ctr Koi NaCl), (iv) salt-treated CEV-infected koi (CEV Koi NaCl), (v) control Amur sazan (Ctr Amur), and (vi) CEV-infected Amur sazan (CEV Amur). Fish from each group were placed in plastic tanks (120 L) with aeration at 20°C. Before infection, the water temperature was lowered from 20°C to 18°C by 1°C per day in the case of experiments performed at 18°C, and from 20°C to 12°C by 1°C per day in the case of experiments performed at 12°C. Fish from each group were acclimatized to the final water temperatures for at least 2 weeks. CEV infection was performed by adding to each tank KSD-affected virus-shedding donor fish (n = 3 fish per infected tank) or healthy, non-infected fish (n = 3 fish per control tank). In the salt-treated koi groups, NaCl (0.6% w/v) was added to the water on day 3 post exposure. At 6 days post exposure, fish from each group were immediately transferred all at once to a tank with an overdose of a buffered solution of MS-222 in water (0.4 g/L) and were euthanized prior to blood and tissues collection.

### 2.5 Sample collection

Blood was collected from the caudal vein using S-Monovette (Sarstedt, Germany). Blood, 100 µl, was used for blood parameter analysis, while the rest of the blood was centrifuged at 2,400 × *g* for 10 min at 4°C. Plasma was collected and kept at −80°C for further analysis. The brain was removed, and the hypothalamus containing the nucleus preopticus (NPO) was carefully separated from the brain. The pituitary gland (PIT), located at the base of the brain, was collected after removing the brain. Head kidney (HK) and gills were also collected. All tissues were placed into RNAlater and kept at −80°C for further analysis.

### 2.6 Blood parameters analysis

Blood, 100 µl, was collected into a glass capillary and loaded into an OPTI CCA-TS blood gas analyzer (OPTI Medical Systems, USA) equipped with OPTI sensor cassettes E-Ca for measuring blood pH, carbon dioxide partial pressure (pCO_2_), oxygen partial pressure (pO_2_), base excess (BE), total carbon dioxide (tCO_2_), blood hydrogen carbonate (HCO_3_), standard hydrogen carbonate (CO_2_-independent) (stHCO_3_), sodium (Na^+^), potassium (K^+^), and calcium (Ca^2+^) levels, as well as the total hemoglobin (tHb) content and hematocrit (Hct). Blood parameters were measured as described previously (14).

### 2.7 Glucose and cortisol concentration

Blood plasma was used to assess glucose and cortisol concentration. Glucose concentration was measured using glucose test strips and glucometer iXell® OLED (Genexo, Poland). Cortisol concentration was measured using immunoenzymatic assay according to the manufacturer’s protocol with commercial kit Cortisol ELISA (Neogen, USA).

### 2.8 Gill histopathology

From each fish, a gill arch was collected immediately after killing and fixed with 4% phosphate-buffered formaldehyde solution for subsequent histological analysis. Sections (3 μm) of paraffin-embedded gills were cut and stained with alcian blue and periodic acid Schiff (AB-PAS). Pathomorphological changes of the gill tissue caused by CEV infection were assessed using the methodology described earlier (33). The following changes were recorded: hyperplasia of epithelial cells, lifting of the epithelium, proliferation of the interlamellar cellular mass, the presence of cells with edema or foamy contents, and the presence of infiltrating granulocytes.

### 2.9 Immunofluorescent staining and imaging

Gill tissues fixed with 4% phosphate-buffered formaldehyde and paraffin embedded were sectioned at 4 µm and placed on adhesion slides Superfrost Plus Gold (Epredia, UK) and stained as described previously (34). Briefly, prepared slides were submitted to deparaffinization, antigen retrieval with sodium citrate buffer (10 mM of sodium citrate, 0.05% Tween 20, pH = 6), and blocking with SuperBlock Blocking Buffer (Thermo Fisher Scientific, USA). Next, slides were incubated with a primary antibody: anti-ZAP70 rabbit monoclonal antibody (Zap-70 (99F2) (35), #2705, Cell Signaling Technology, Inc., USA) at 1:300 in Pierce Immunostain Enhancer (Thermo Fisher Scientific, USA). After washing, slides were incubated with a secondary antibody: Alexa Fluor 555-labeled donkey anti-rabbit antibody (Thermo Fisher Scientific, USA) at 1:1,000 in Pierce Immunostain Enhancer (Thermo Fisher Scientific, USA). Slides were washed and mounted with Roti-Mount with DAPI (ROTH, Germany). Images were captured on KEYENCE BZ-X810 (Keyence, Japan) (×20 objective magnification) and analyzed using ImageJ. Only ZAP70 positive cells associated with the secondary lamella were counted using digitally magnified images. The following procedure was used for counting ZAP70-positive cells: (i) counting the secondary lamellae, (ii) counting the positive cells on previously counted lamellae, and (iii) counting the ratio of ZAP70-positive cells to the number of secondary lamellae.

### 2.10 DNA isolation

DNA was isolated from gill tissue stored in *RNAlater*. First, the tissue was mechanically lysed in a Tissuelyser II (Qiagen, Germany), and DNA was extracted using the QIAamp DNA Mini Kit (Qiagen, Germany) according to the manufacturer’s protocol. After isolation, samples were diluted to 50 ng μl^−1^ and stored at −80°C.

### 2.11 RNA isolation and cDNA synthesis

Total RNA isolation and synthesis of cDNA was performed as previously described (5, 36). In order to verify the purity of the RNA, selected samples without the addition of reverse transcriptase (no-RT) were used to check for genomic DNA contamination. cDNA samples were diluted 40× with nuclease-free water (Thermo Fisher Scientific, Germany).

### 2.12 Real-time quantitative PCR

#### 2.12.1 Viral load analysis

For monitoring the viral load, DNA was isolated from gills, and the gene encoding the P4a capsid core protein of CEV (p4a) was used as a target (**Supplementary Table S1**). For quantification of viral load, an SYBR Green-based RT-qPCR was used as described earlier (14). Viral load is presented as normalized copy number.

#### 2.12.2 Gene expression analysis

Expression of stress-related genes (32), immune-related genes selected for validation of the multiplex RT-qPCR results (14), and genes encoding ZAP70 (35) and CD3 (37) was studied using RotorGene Q instrument (Qiagen). Gene expression analyses were performed on a cDNA template as previously described (5, 36). Each reaction was performed in duplicate. No-RT samples and non-template controls were run in selected plates. Gene expression changes are presented as a ratio of reference genes encoding the 40S ribosomal protein S11 (*40s*) and the elongation factor 1 alpha (*ef1a*) to target genes (**Supplementary Table S1**) using the Pfaffl method in accordance to the equation:


$$\text{Ratio}=\text{E}\hat{\;}\text{Ct Reference}/\text{E}\hat{\;}\text{Ct Target}$$

where E is the amplification efficiency, and Ct is the threshold cycle.

Expression of immune-related genes was studied using multiplex RT-qPCR at Biomark instrument. The integrated fluidic circuit (IFC) technology of Standard BioTools was used to profile the expression of 31 target genes and 4 reference genes (38) (**Supplementary Table S1**). For these multiplex RT-qPCR analyses, 48.48 Gene Expression IFC chips (Standard BioTools) were used with the BioMark HD system (Standard BioTools). First, 10 ng/μl of RNA extracted from the gills were reverse-transcribed (42°C, 30 min) using the Reverse Transcription Master Mix (Standard BioTools). The resulting cDNA was mixed with primers (100 μM) and the PreAmp master mix (Standard BioTools) and preamplified in 13 cycles (95°C, 15 s; 60°C, 4 min) in a TAdvanced thermocycler (Biometra). Exonuclease I (New England BioLabs) was added to degrade single-stranded oligonucleotide primers for 30 min at 37°C. After the addition of 43 μl of TE buffer (Sigma), the cDNA samples were diluted in SsoFast EvaGreen Supermix with Low ROX (Bio-Rad) and 20× DNA Binding Dye Sample Loading Reagent (Standard BioTools). For the priming of the 48.48-IFC chips in the MX Controller (Standard BioTools), the primers and sample mixes were transferred to the assay and sample inlets on the 48.48-IFC chip. Finally, multiplex RT-qPCR was performed according to the manufacturer’s protocol “GE Fast 48x48 PCR+Melt v2.pcl.” Results were analyzed with the Standard BioTools RealTime PCR Analysis Software v. 4.5.2 and normalized against the geometric mean of the reference genes *actb*, *gapdh*, *eef1a1*, and *rps11*. Heatmaps of gene expression were created with Heatmapper (39).

### 2.13 Statistical analysis

Statistical analysis was carried out in a SigmaPlot 12.5 software (Systat Software). Data from viral load and gene expression studies were transformed using a Log10(x) transformation before statistical analysis. Significant differences (p ≤ 0.05) in viral load and in gene expression, cortisol, and glucose concentration were assessed using two-way ANOVA, with subsequent pairwise multiple comparisons using the Holm–Sidak test.

## 3 Results

### 3.1 Stress response after restraint stress

**Figure 1:**
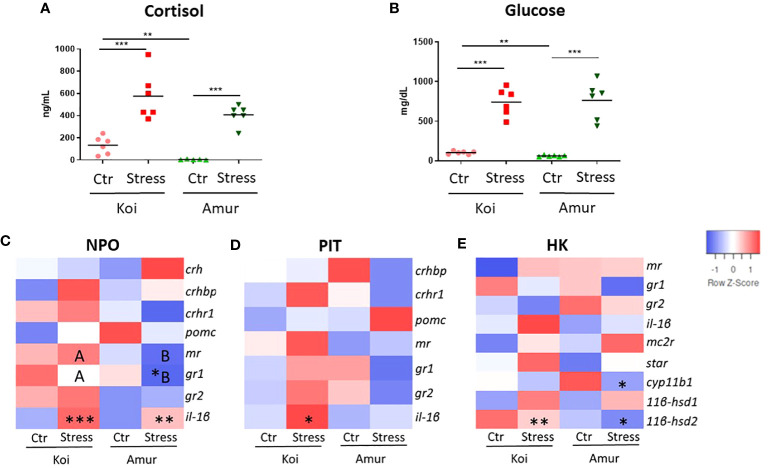
Level of **(A)** cortisol (ng/mL) and **(B)** glucose (mg/dL) in blood plasma of unstressed (Ctr) and stressed (Stress) koi and Amur sazan. Heatmaps showing changes in the constitutive expression of stress-involved genes revealed by RT-qPCR in: **(C)** hypothalamic nucleus preopticus (NPO), **(D)** pituitary gland (PIT), and **(E)** head kidney (HK) of unstressed and stressed koi and Amur sazan. Asterisks indicate statistically significant differences between the studied group **(A, B)** or between unstressed and stressed fish within the strain **(C–E)** (*p ≤ 0.05; **p ≤ 0.01; ***p ≤ 0.001). Different capital letters (e.g., A vs. B) indicate statistically significant differences at p ≤ 0.05 between stressed koi and stressed Amur sazan **(C–E)**. Statistical analysis was performed by two-way ANOVA with subsequent pairwise multiple comparisons using the Holm–Sidak test. The data are presented as n = 6 individual values with the mean indicated as a horizontal line **(A, B)** or as the mean of n = 6 fish **(C–E)**.

To compare the stress response between koi carp and Amur sazan, fish were subjected to the prolonged restraint stress protocol, and the cortisol and glucose levels in blood plasma as well as the expression of genes involved in the activation of the HPI axis were studied. As expected, restraint stress induced significant increase in the plasma levels of cortisol (from 134 to 575 ng/ml in koi and from 4 to 407 ng/ml in Amur sazan) and glucose (from 104 to 742 mg/dl in koi and from 62 to 763 mg/dl in Amur sazan). Interestingly, basal levels of cortisol and glucose in blood plasma were significantly higher in control koi than in control Amur sazan (**Figures 1A, B**).

Upon restraint stress, the gene expression study revealed an upregulated expression of *il-1β* in NPO of both koi and Amur sazan and in PIT of koi carp. Moreover, *gr1* expression was downregulated in NPO of Amur sazan (**Figures 1C, D**; **Supplementary Table S2**). In HK of both carp strains, the restraint stress induced expression of the *11βhsd2* gene (encoding 1β-hydroxysteroid dehydrogenase type 2 converting active cortisol into inactive cortisone), while in HK of Amur sazan, the restraint stress downregulated the expression of the *cyp11b1* gene (encoding 1β-hydroxylase involved in conversion of cortisol from 11-deoxycortisol) in HK (**Figure 1E**; **Supplementary Table S2**).

No statistically significant differences were observed between unstressed koi and unstressed Amur (**Figures 1C–E**; **Supplementary Table S2**). The only genes that differed significantly in expression level between stressed koi carp and Amur sazan were *gr1* and *mr* in NPO. The expression of both genes was significantly higher in stressed koi compared to stressed Amur sazan (**Figure 1C**; **Supplementary Table S2**). Thus, our study demonstrated similar responses of koi and Amur sazan to restraint stress, although the basal levels of plasma cortisol and glucose were higher in koi carp.

### 3.2 Clinical signs of KSD

There were clear differences in KSD development between 12°C and 18°C. At 12°C, only CEV-infected koi showed mild clinical signs of infection (slight lethargy) at day 6 post exposure to CEV-positive donors, while CEV-infected salt-treated koi and Amur sazan did not display any clinical signs of disease. At 18°C, CEV-infected koi started to demonstrate clinical signs of KSD at 5 days post exposure. At 6 days post exposure, fish showed severe lethargy, were laying at the bottom of the tank on the side of their body, and were not responsive to stimuli. CEV-infected salt-treated koi and Amur sazan as well as uninfected control fish did not display any clinical signs of KSD.

### 3.3 Gill morphology during CEV infection

**Figure 2:**
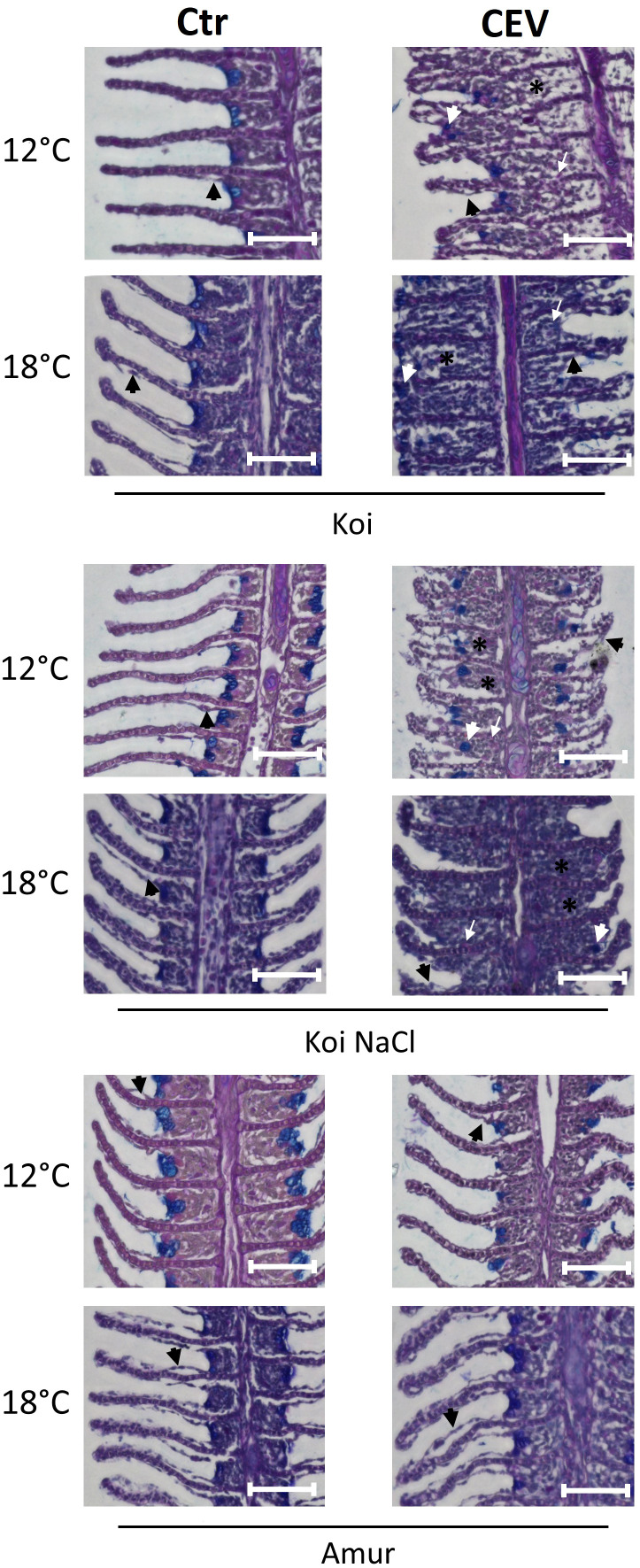
Histology of the gills during CEV infection (6 days post exposure). Black arrowheads indicate examples of lifting of the epithelial cells on the secondary lamella. White arrowheads indicate examples of translocated goblet cells. Black (*) symbol indicates examples of occluded interlamellar space. White arrows indicate examples of macrophage infiltration. Bar 100 µm. AB-PAS staining.

Gill tissues collected at 6 days post exposure were studied for histopathological changes. At 12°C, gills of CEV-infected koi showed partial occlusion of the intralamellar space, most likely related to hyperplasia of epithelial cells of the gills, and low infiltration of eosinophilic cells were noted. CEV-infected salt-treated koi showed similar histopathological changes, while Amur sazan and control uninfected fish did not show any obvious gill pathology. At 18°C, nearly complete occlusion of the intralamellar space with the accumulation of cellular debris and an infiltration of eosinophilic cells were noted in the gills of CEV-infected koi at 6 days post exposure. Despite showing no visible clinical signs of KSD, salt-treated CEV-infected koi showed almost as severe histopathological changes as CEV-infected koi. In Amur sazan and uninfected controls, no histopathological changes were noted in the gills (**Figure 2**).

### 3.4 Viral load during CEV infection

**Figure 3:**
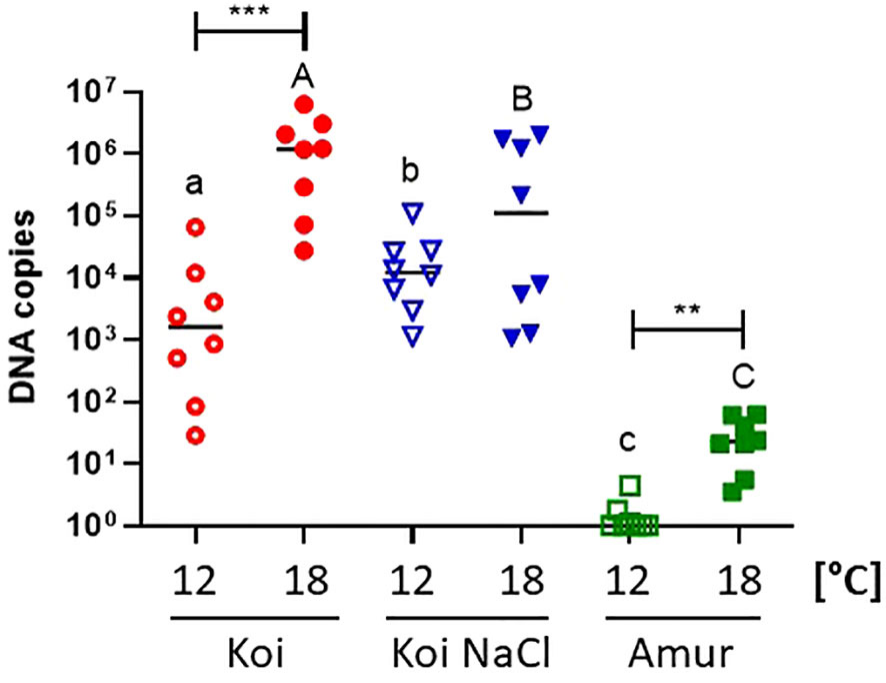
Viral load in the gills of three groups of CEV-infected carp: koi (Koi), salt-treated koi (Koi NaCl), and Amur sazan (Amur), kept at 12°C and 18°C (6 days post exposure). Asterisks indicate statistically significant differences between fish kept at 12°C and 18°C within the strain/group (**p ≤ 0.01; ***p ≤ 0.001). Different lowercase letters (e.g., a vs. b) indicate statistically significant differences at p ≤ 0.05 between the groups of infected fish kept at 12°C. Different capital letters (e.g., A vs. B) indicate statistically significant differences at p ≤ 0.05 between the groups of infected fish kept at 18°C. Statistical analysis was performed using two-way ANOVA with subsequent pairwise multiple comparisons using the Holm–Sidak test. The data are presented as n = 8 individual values with the mean indicated as a horizontal line.

Quantification of CEV at 6 days post exposure revealed significantly higher viral load in the gills of koi and Amur sazan kept at 18°C than in fish kept at 12°C (**Figure 3**). The same trend was also observed in salt-treated koi, although the differences were not statistically significant. At 18°C, the highest viral load was demonstrated in koi carp (mean 1.77 × 10^6^ copies) followed by salt-treated koi (mean 1.64 × 10^5^ copies), while the lowest viral load was measured in Amur sazan (mean 2.9 × 10^1^) (**Figure 3**). These results demonstrate the effect of temperature and carp genetic background on the viral load.

### 3.5 Blood parameters during CEV infection

Chemical parameters were analyzed in the blood of uninfected controls and CEV-infected (at 6 days post exposure to KSD-affected donor koi) koi, salt-treated koi, and Amur sazan, at both 12°C and 18°C. Blood parameters of koi and salt-treated koi were strongly affected by CEV infection at both 12°C (**Table 1**) and 18°C (**Table 2**). At 12°C CEV-infected koi showed increased levels of pH, BE, tCO_2_, HCO_3_, and stHCO_3_ and decrease in pO_2_, Na^+^, and Ca^2+^. In turn, in salt-treated koi, CEV elevated the pH, pCO_2_, BE, tCO_2_, HCO_3_, and stHCO_3_, and decreased pO_2_, Na^+^, K^+^, and Ca^2+^ were measured. In the blood of CEV-infected Amur sazan kept at 12°C, the level of the analyzed parameters was not significantly different compared to uninfected fish, except for the pCO_2_, which was lower in the CEV-infected fish than in uninfected controls (**Table 1**).

**Table 1:** Changes in the blood parameters of uninfected (Ctr) and CEV-infected (CEV) fish from three groups of carp: koi (Koi), salt-treated koi (Koi NaCl), and Amur sazan (Amur) kept at 12°C (6 days post exposure)^1^.

Factor	Koi		Koi NaCl		Amur	
	Ctr	CEV	Ctr	CEV	Ctr	CEV
**pH**	7.36 ± 0.04	7.60 ± 0.11***^A^	7.30 ± 0.08	7.49 ± 0.11***^B^	7.26 ± 0.11	**7.35 ± 0.07^B^**
**pCO_2_** (mmHg)	**26.44 ± 1.61^a^**	27.49 ± 3.87	**26.91 ± 3.04^a^**	30.75 ± 3.80***	**31.04 ± 5.02^b^**	26.83 ± 2.41***
**pO_2_** (mmHg)	36.48 ± 11.10	24.81 ± 4.61* ^A^	44.28 ± 8.80	24.64 ± 4.58***^A^	45.20 ± 25.68	**40.49 ± 11.15^B^**
**BE** (mmol/L)	−9.56 ± 1.66	5.51 ± 7.84*^A^	−12.36 ± 3.24	−0.69 ± 4.25*^AB^	−12.19 ± 3.47	**−10.29 ± 2.39^B^**
**tCO_2_** (mmol/L)	15.44 ± 1.24	27.96 ± 6.66***^A^	13.79 ± 2.37	23.43 ± 3.03***^A^	14.66 ± 2.14	**15.20 ± 1.46^B^**
**HCO_3_** (mmol/L)	14.65 ± 1.20	27.13 ± 6.64***^A^	12.98 ± 2.37	22.51 ± 3.06***^A^	13.71 ± 2.15	**14.40 ± 1.48^B^**
**stHCO_3_** (mmol/L)	16.75 ± 1.29	29.23 ± 6.64***^A^	14.66 ± 2.44	23.91 ± 3.52***^B^	14.84 ± 2.58	**16.21 ± 1.85^C^**
**Na^+^** (mmol/L)	143.84 ± 2.67	113.44 ± 11.08***^A^	147.08 ± 3.35	131.98 ± 5.59***^B^	144.39 ± 2.38	**145.26 ± 1.13^C^**
**K^+^** (mmol/L)	2.82 ± 0.25	**3.21 ± 0.79^A^**	3.05 ± 0.40	2.61 ± 0.27*^B^	2.98 ± 0.42	**2.96 ± 0.38^AB^**
**Ca^2+^** (mmol/L)	**1.24 ± 0.16^a^**	1.13 ± 0.09*^A^	**1.35 ± 0.04^b^**	1.21 ± 0.06**^AB^	**1.22 ± 0.09^a^**	**1.25 ± 0.09^B^**
**tHB** (g/dL)	5.44 ± 0.38	6.90 ± 1.81	6.68 ± 1.18	6.67 ± 0.85	7.06 ± 1.60	6.40 ± 0.79
**Hct(c)** (%)	16.34 ± 1.11	20.67 ± 5.40	20.03 ± 3.51	20.00 ± 2.52	21.16 ± 4.82	19.23 ± 2.37

^1^Asterisks indicate statistically significant differences between uninfected and CEV-infected fish within the stain/group (*p ≤ 0.05; **p ≤ 0.01; ***p ≤ 0.001). Different lowercase letters (e.g., a vs. b) indicate statistically significant differences at p ≤ 0.05 between the groups of uninfected fish. Different capital letters (e.g., A vs. B) indicate statistically significant differences at p ≤ 0.05 between the groups of CEV-infected fish. Statistically significant results are indicated in bold. Statistical analysis was performed using two-way ANOVA with subsequent pairwise multiple comparisons using the Holm–Sidak test. The data are the means of n = 8 fish.

**Table 2:** Changes in blood parameters of uninfected (Ctr) and CEV-infected (CEV) fish from three groups of carp: koi (Koi), salt-treated koi (Koi NaCl), and Amur sazan (Amur) kept at 18°C (6 days post exposure)^1^.

Factor	Koi		Koi NaCl		Amur	
	Ctr	CEV	Ctr	CEV	Ctr	CEV
**pH**	7.21 ± 0.10	7.53 ± 0.17***^A^	7.16 ± 0.07	7.32 ± 0.10**^B^	7.17 ± 0.06	**7.16 ± 0.08 ^C^**
**pCO_2_** (mmHg)	**27.90 ± 3.11 ^ab^**	**28.64 ± 4.13 ^A^**	**25.41 ± 1.26 ^a^**	29.94 ± 4.15** ^A^	**30.94 ± 1.36 ^b^**	**33.69 ± 3.15 ^B^**
**pO_2_** (mmHg)	33.24 ± 10.86	21.46 ± 7.70	41.95 ± 16.21	25.21 ± 8.97**	44.21 ± 15.23	33.21 ± 7.47
**BE** (mmol/L)	−15.55 ± 3.63	1.98 ± 9.85*^A^	−18.30 ± 2.66	−9.54 ± 6.25*^AB^	−16.30 ± 2.10	**−15.90 ± 2.82 ^B^**
**tCO_2_** (mmol/L)	11.85 ± 2.36	25.06 ± 7.75***^A^	9.66 ± 1.69	16.54 ± 5.24***^B^	11.91 ± 1.30	**12.69 ± 1.58^C^**
**HCO_3_** (mmol/L)	11.01 ± 2.35	24.20 ± 7.79***^A^	8.88 ± 1.64	15.60 ± 5.15***^B^	10.99 ± 1.30	**11.66 ± 1.62^C^**
**stHCO_3_** (mmol/L)	12.50 ± 2.48	26.30 ± 8.19***^A^	10.49 ± 1.74	16.90 ± 4.76***^B^	11.96 ± 1.35	**12.38 ± 1.87 ^C^**
**Na^+^** (mmol/L)	136.64 ± 2.42	99.00^#^ ± 0.00***^A^	139.83 ± 3.40	133.75 ± 10.59* ^B^	136.54 ± 1.75	**137.81 ± 1.30 ^B^**
**K^+^** (mmol/L)	3.72 ± 1.91	4.42 ± 0.86***^A^	3.58 ± 1.14	**2.51 ± 0.65 ^B^**	2.87 ± 0.36	**4.79 ± 6.32 ^B^**
**Ca^2+^** (mmol/L)	1.35 ± 0.06	0.93 ± 0.21*^A^	1.33 ± 0.08	**1.28 ± 0.15 ^AB^**	1.32 ± 0.05	**2.57 ± 3.55 ^B^**
**tHB** (g/dL)	8.61 ± 1.26	11.35 ± 2.44***^A^	7.56 ± 0.93	**8.11 ± 1.43^B^**	8.26 ± 0.87	9.84 ± 1.36*^C^
**Hct(c)** (%)	25.93 ± 3.80	34.08 ± 7.30***^A^	22.64 ± 2.75	**24.39 ± 4.32 ^B^**	24.90 ± 2.59	29.60 ± 4.12*^C^

^1^Asterisks indicate statistically significant differences between uninfected and CEV-infected fish within the strain/group (*p ≤ 0.05; **p ≤ 0.01; ***p ≤ 0.001). Different lowercase letters (e.g., a vs. b) indicate statistically significant differences at p ≤ 0.05 between the groups of uninfected fish. Different capital letters (e.g., A vs, B) indicate statistically significant differences at p ≤ 0.05 between the groups of CEV-infected fish. Statistically significant results are indicated in bold. Statistical analysis was performed using two-way ANOVA, with subsequent pairwise multiple comparisons using the Holm–Sidak test. The data are the means of n = 8 fish.

^#^sodium was below detection limit which was 100 mmol/L.

CEV infection at 18°C resulted in increased pH, BE, tCO_2_, HCO_3_, stHCO_3_, K^+^, tHB, and Hct(c) and decreased Na^+^ and Ca^2+^ concentrations in koi. Salt-treated koi infected with CEV showed higher levels of pH, pCO_2_, BE, tCO_2_, HCO_3_, and stHCO_3_ and a decrease in pO_2_ and Na^+^ than the uninfected control group. Increased levels of tHB and Hct(c) were the only changes in blood parameters of infected Amur sazan (**Table 2**).

### 3.6 Stress response during CEV infection

**Figure 4:**
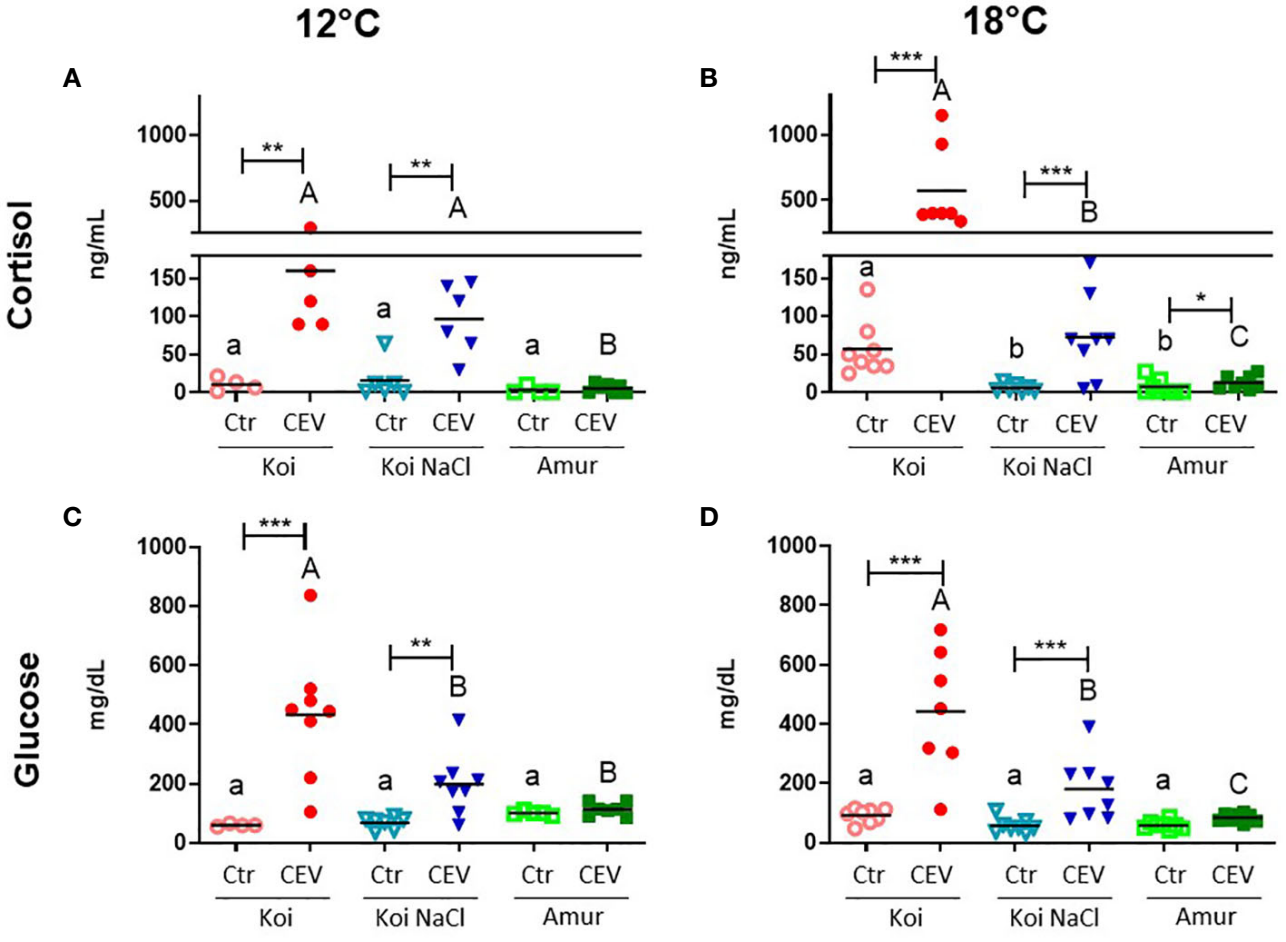
Level of **(A, B)** cortisol and **(C, D)** glucose in the blood plasma of uninfected (Ctr) and CEV-infected (CEV) fish from three groups of carp: koi (Koi), salt-treated koi (Koi NaCl), and Amur sazan (Amur) kept at 12°C or 18°C (6 days post exposure). Asterisks indicate statistically significant differences between uninfected and CEV-infected within the strain/group (*p ≤ 0.05; **p ≤ 0.01; ***p ≤ 0.001). Different lowercase letters (e.g., a vs. b) indicate statistically significant differences at p ≤ 0.05 between the groups of uninfected fish. Different capital letters (e.g., A vs. B) indicate statistically significant differences at p ≤ 0.05 between the groups of CEV-infected fish. Statistical analysis was performed using two-way ANOVA with subsequent pairwise multiple comparisons using the Holm-Sidak test. The data are presented as n = 5–8 individual values with the mean indicated as a horizontal line.

To study the effect of CEV infection on stress response in koi carp and Amur sazan, cortisol and glucose levels were measured in blood plasma. In koi carp kept at 12°C, infection with CEV resulted in significantly increased cortisol concentration (from 11 to 160 ng/ml in koi and from 16 to 97 ng/ml in salt-treated koi) and in increased glucose concentration (from 61 to 434 mg/dl in koi, and from 69 to 198 mg/dl in salt-treated koi). In contrast, there were no significant differences in the cortisol and glucose concentrations between CEV-infected and control Amur sazan (**Figures 4A, C**). At 18°C, cortisol and glucose levels were significantly higher in all infected groups of fish compared to uninfected control fish, with the exception of glucose level measured in the blood of Amur sazan. In all strains/groups of fish kept at 18°C, infection with CEV resulted in increased cortisol concentration (from 57 to 573 ng/ml in koi, from 6 to 72 ng/ml in salt-treated koi, and from 7 to 13 ng/ml in Amur sazan) and in increased glucose concentration (from 92 to 442 mg/dl in koi and from 57 to 180 mg/dl in salt-treated koi) (**Figures 4B, D**).

In the CEV-infected fish, significantly higher cortisol and glucose concentrations were observed in koi carp compared to salt-treated koi and Amur sazan at both temperatures, except for cortisol level at 12°C, where no significant differences were observed between koi and salt-treated koi carp. At 18°C, but not at 12°C, the basal cortisol level in uninfected fish was significantly higher in koi compared to salt-treated koi and Amur sazan (**Figure 4B**). There were no significant differences in the basal concentration of glucose between uninfected fish from different strains/treatment at both temperatures (**Figures 4C, D**).

**Figure 5:**
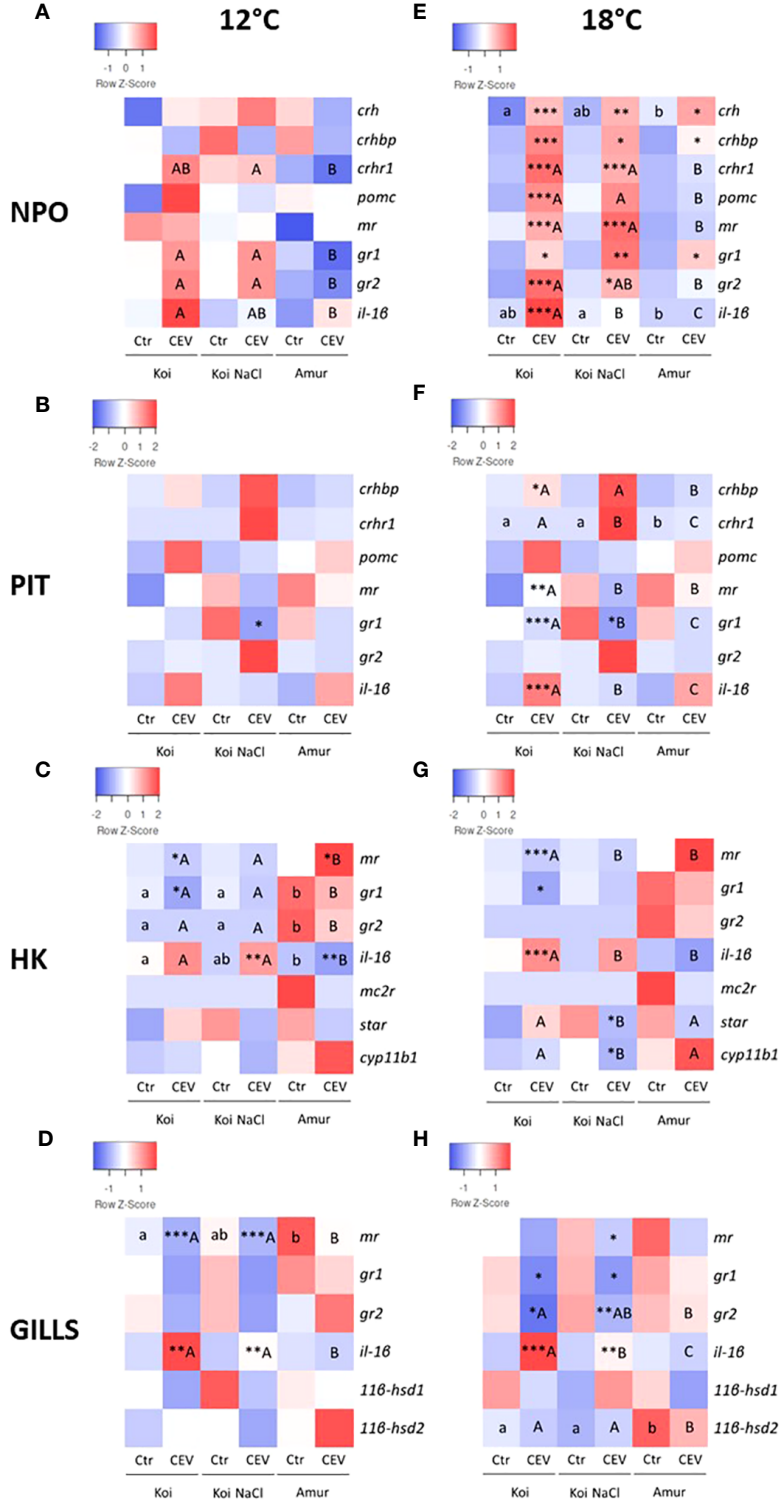
Heatmaps showing changes in the constitutive expression of stress-involved genes revealed by RT-qPCR in **(A, E)** hypothalamic nucleus preopticus (NPO), **(B, F)** pituitary gland (PIT), **(C, G)** head kidney (HK), and **(D, H)** gills of uninfected (Ctr) and CEV-infected (CEV) fish from three groups of carp: koi (Koi), salt-treated koi (Koi NaCl), and Amur sazan (Amur) kept at 12°C **(A–D)** and 18°C **(E–H)** (6 days post exposure). Asterisks indicate statistically significant differences between uninfected and CEV-infected fish within the strain/group (*p ≤ 0.05; **p ≤ 0.01; ***p ≤ 0.001). Different lowercase letters (e.g., a vs. b) indicate statistically significant differences at p ≤ 0.05 between the strains/groups of uninfected fish. Different capital letters (e.g., A vs. B) indicate statistically significant differences at p ≤ 0.05 between the groups of CEV-infected fish. Statistical analysis was performed using two-way ANOVA with subsequent pairwise multiple comparisons using the Holm-Sidak test. The data are the mean of n = 8 fish.

The CEV-induced stress response was also studied by analyzing the expression of genes involved in the stress response in the HPI axis organs and in the gills of fish kept at 12°C and 18°C. At 12°C, no significant changes in gene expression after CEV infection were observed in NPO and PIT in koi, salt-treated koi, and Amur sazan, with the exception of *gr1*, which was downregulated in PIT of CEV-infected salt treated koi, compared to uninfected control salt-treated koi (**Figures 5A, B**; **Supplementary Table S3**). In HK, CEV infection downregulated *mr* and *gr1* expression in koi, upregulated *il-1β* expression in salt-treated koi, and upregulated *mr* and downregulated *il-1β* expression in Amur sazan at 12°C (**Figure 5C**; **Supplementary Table S3**). In the gills, CEV infection induced *il-1β* and reduced *mr* expression in koi and salt-treated koi at 12°C (**Figure 5D**; **Supplementary Table S3**).

At 18°C, CEV infection induced significantly the expression of all examined stress-related genes in NPO of koi and salt-treated koi (with the exceptions of *pomc* and *il-1β* in the salt-treated group), whereas only a slight upregulation of *crh*, *crhbp*, and *gr1* expression was observed in Amur sazan (**Figure 5E**; **Supplementary Table S4**). In PIT of fish kept at 18°C, CEV infection induced significant upregulation of the expression of *crhbp* and *il-1β* and downregulation of *mr* and *gr1* in the case of koi and downregulation of *gr1* in salt-treated koi. No significant changes in gene expression between control and infected fish were observed in Amur sazan (**Figure 5F**; **Supplementary Table S4**). In HK, upregulation of *il-1β* and downregulation of *mr* and *gr1* expression was observed in CEV-infected koi at 18°C. In salt-treated koi, CEV infection downregulated expression of the *star* gene (encoding steroidogenic acute regulatory protein involved in cholesterol transport to mitochondria) and *cyp11b1* (encoding cytochrome P450 family 11 subfamily B member 1 protein involved in steroid synthesis). No significant changes in gene expression were observed in HK between control and infected Amur sazan kept at 18°C (**Figure 5G**; **Supplementary Table S4**). In the gills, CEV infection in fish kept at 18°C reduced *gr1* and *gr2* expression and induction of *il-1β* expression in koi and salt-treated koi. Moreover, downregulation of *mr* expression was observed in infected salt-treated koi. No significant changes in gene expression were observed between control and infected Amur sazan kept at 18°C (**Figure 5H**; **Supplementary Table S4**). Together, our results clearly demonstrated that CEV infection induces stress response, which is more pronounced in susceptible koi fish at 18°C, and this also correlates with the results of the viral load analysis.

### 3.7 Immune response during CEV infection

**Figure 6:**
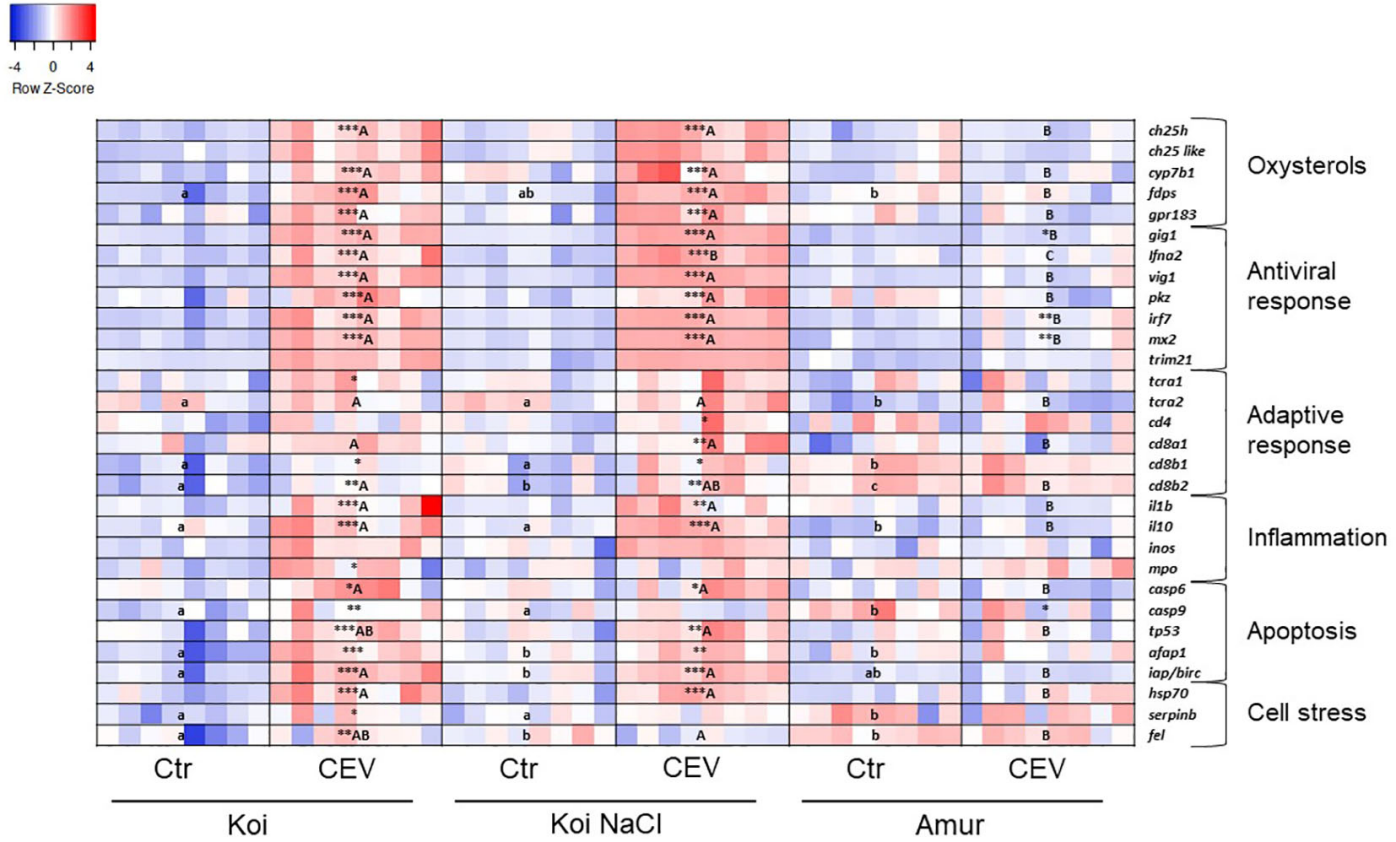
Heatmaps showing changes in gene expression of immune-related genes revealed by nanoscale RT-qPCR in the gills of uninfected (Ctr) and CEV-infected (CEV) fish from three groups of carp: koi (Koi), salt-treated koi (Koi NaCl), and Amur sazan (Amur) kept at 12°C (6 days post exposure). Asterisks indicate statistically significant differences between uninfected and CEV-infected within the strain/group (*p ≤ 0.05; **p ≤ 0.01; ***p ≤ 0.001). Different lowercase letters (e.g., a vs. b) indicate statistically significant differences at p ≤ 0.05 between the groups of uninfected fish. Different capital letters (e.g., A vs. B) indicate statistically significant differences at p ≤ 0.05 between the strains/groups of CEV-infected fish. Statistical analysis was performed using two-way ANOVA with subsequent pairwise multiple comparisons using the Holm–Sidak test. For each group, n = 8 fish are presented. One square equals data from one individual fish.

**Figure 7:**
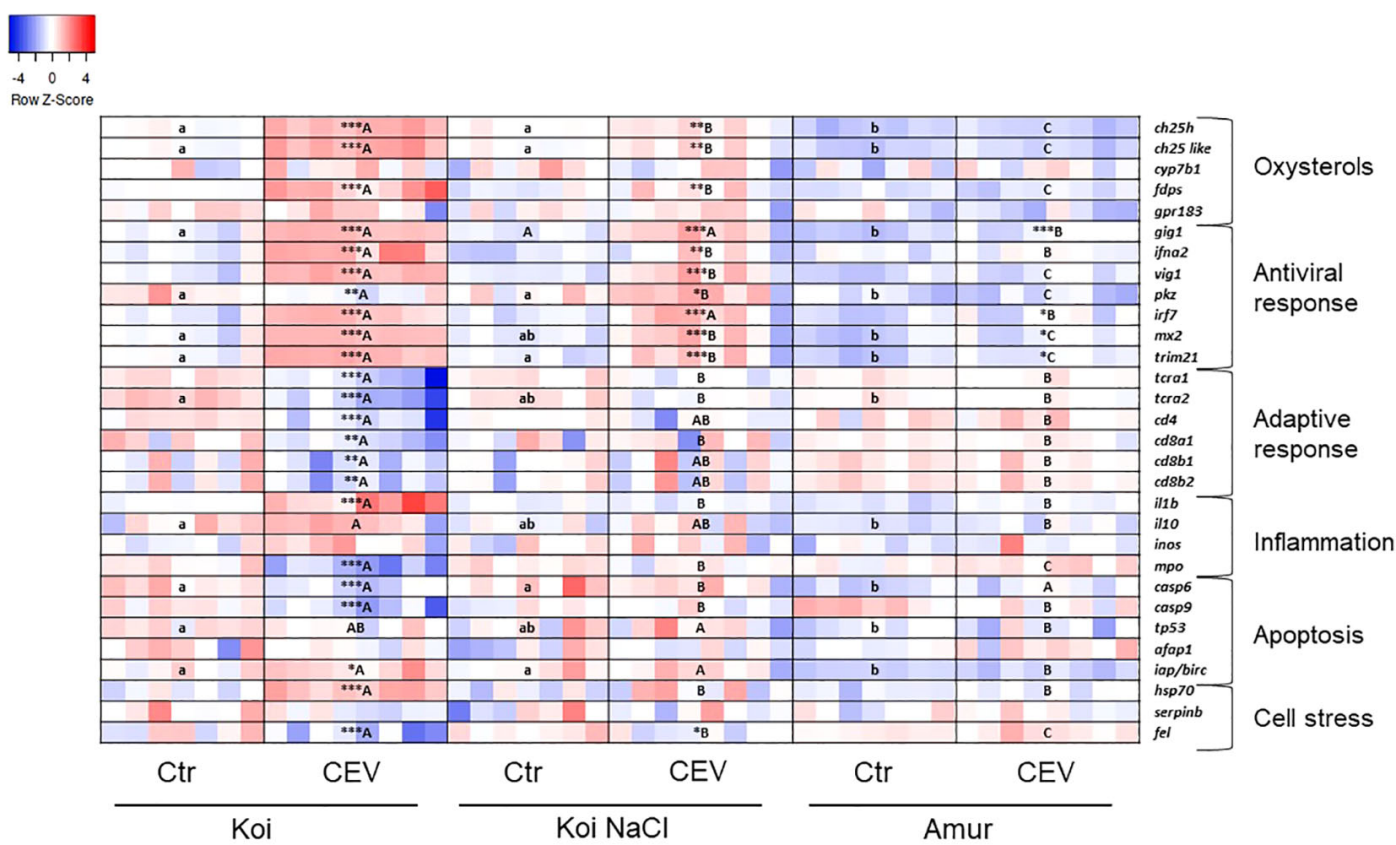
Heatmaps showing changes in gene expression of immune-related genes revealed by nanoscale RT-qPCR in gills of uninfected (Ctr) and CEV-infected (CEV) fish from three groups of carp: koi (Koi), salt-treated koi (Koi NaCl), and Amur sazan (Amur) kept at 18°C (6 days post exposure). Asterisks indicate statistically significant differences between uninfected and CEV-infected within the strain/group (*p ≤ 0.05; **p ≤ 0.01; ***p ≤ 0.001). Different lowercase letters (e.g., a vs. b) indicate statistically significant differences at p ≤ 0.05 between the strains/groups of uninfected fish. Different capital letters (e.g., A vs. B) indicate statistically significant differences at p ≤ 0.05 between the strains/groups of CEV-infected fish. Statistical analysis was performed using two-way ANOVA, with subsequent pairwise multiple comparisons using the Holm–Sidak test. For each group, n = 7–8 fish are presented. One square equals data from one individual fish.

Multiplex RT-qPCR enabled the analysis of the expression of genes involved in the oxysterol pathway, antiviral response, adaptive immune response, inflammation, apoptosis, and cell stress in CEV-infected (at 6 days post exposure of fish to CEV-infected donors) and control fish. The results presented in a heatmap (**Figures 6**, **7**) were later confirmed using RT-qPCR with selected genes involved in antiviral and adaptive immune response.

In koi and salt-treated koi, CEV infection at 12°C induced the expression of genes involved in the oxysterol pathway (*ch25h*, *cyp7b1*, *fdps*, and *gpr183*). Expression of antiviral response genes (*gig*, *ifna2*, *vig*, *pkz*, *irf7*, and *mx2*) was upregulated in CEV-infected koi and infected salt-treated koi. Expression of some of these antiviral genes (*gig*, *irf7*, and *mx2*) was also upregulated in Amur sazan. CEV infection induced upregulation of the expression of selected adaptive-immune genes in koi (*tcra1*, *cd8b1*, and *cd8b2*) and in salt-treated koi (*cd4*, *cd8a1*, *cd8b1*, and *cd8b2*). Expression of genes involved in inflammatory response was also upregulated in infected koi (*il-1β*, *il-10*, and *mpo*) and infected salt-treated koi (*il-1β* and *il-10*). In Amur sazan, no upregulation of the expression of adaptive and inflammatory response genes was observed. Moreover, in koi and salt-treated koi, CEV infection upregulated the expression of genes encoding proteins involved in apoptosis (*casp6*, *tp53*, *afap1*, and *iap/birc*). Interestingly, *casp9* expression was upregulated upon CEV infection in koi and downregulated in Amur sazan. Expression of genes related to cell stress (*hsp70*, *serpinb1*, and *fel*) was also upregulated in infected koi, while only *hsp70* expression was upregulated in infected salt-treated koi. In Amur sazan, no changes in the expression of cell stress-related genes were demonstrated upon CEV infection (**Figure 6**; **Supplementary Table S5**).

In koi and salt-treated koi, CEV infection performed at 18°C resulted in the upregulation of the expression of genes involved in the oxysterol pathway (*ch25h*, *ch25-like*, and *fdps*). Expression of antiviral genes (*gig1*, *ifna2*, *vig1*, *irf7*, *mx2*, and *trim21*) was upregulated in infected koi and infected salt-treated koi, while the expression of *gig1*, *irf7*, *mx2*, and *trim21* was elevated in infected Amur. Moreover, the expression of *pkz* was upregulated in salt-treated CEV-infected koi, but was downregulated in CEV-infected koi. Interestingly, CEV infection of koi at 18°C resulted in the reduced expression of genes involved in adaptive immune response (*tcra1*, *tcra2*, *cd4*, *cd8a1*, *cd8b1*, and *cd8b2*). Expression of pro-inflammatory *il-1β* was upregulated in koi upon CEV infection, but expression of gene excoding myeloperoxidase (*mpo*) was downregulated in these fish. Expression of apoptotic genes was differently altered by the CEV infection in koi, where expression of *casp6* and *casp9* was downregulated, while *iap/birc* expression was upregulated. During CEV infection at 18°C, *hsp70* expression was significantly upregulated in koi, while expression of *fel* was downregulated in koi and salt-treated koi (**Figure 7**; **Supplementary Table S6**).

**Figure 8:**
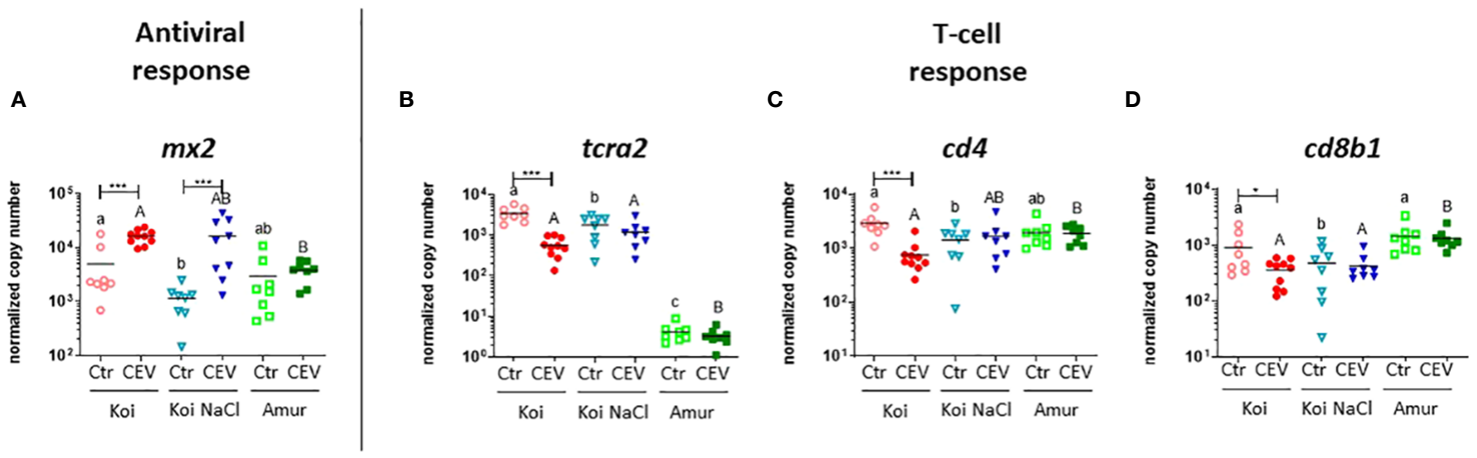
Expression of genes involved in **(A)** antiviral response and **(B–D)** adaptive response revealed by RT-qPCR in the gills of uninfected (Ctr) and CEV-infected (CEV) fish from three groups of carp: koi (Koi), salt-treated koi (Koi NaCl), and Amur sazan (Amur) kept at 18°C (6 days post exposure). Asterisks indicate statistically significant differences between uninfected and CEV-infected individuals within the strain/group (*p ≤ 0.05; *** p≤ 0.001). Different lowercase letters (e.g., a vs. b) indicate statistically significant differences at p ≤ 0.05 between the strains/groups of uninfected fish. Different capital letters (e.g., A vs. B) indicate statistically significant differences at p ≤ 0.05 between the strains/groups of CEV-infected fish. Statistical analysis was performed using two-way ANOVA, with subsequent pairwise multiple comparisons using the Holm–Sidak test. The data are presented as n = 5–9 individual values with the mean indicated as a horizontal line.

RT-qPCR analysis of selected genes was performed to validate the multiplex RT-qPCR results. Our study confirmed the upregulation of the expression of antiviral genes (*mx2*) and downregulation of the expression of adaptive response genes (*tcra2*, *cd4*, and *cd8b1*) in CEV-infected koi at 18°C (**Figures 8A–D**). Similar to the results of multiplex RT-qPCR, expression of *mx2* was also upregulated in CEV-infected salt-treated koi compared to that in control fish (**Figure 8A**), whereas expression of *tcra2*, *cd4*, and *cd8b1* was not changed in CEV-infected salt-treated koi and Amur sazan compared to that in control fish. (**Figures 8B–D**).

**Figure 9:**
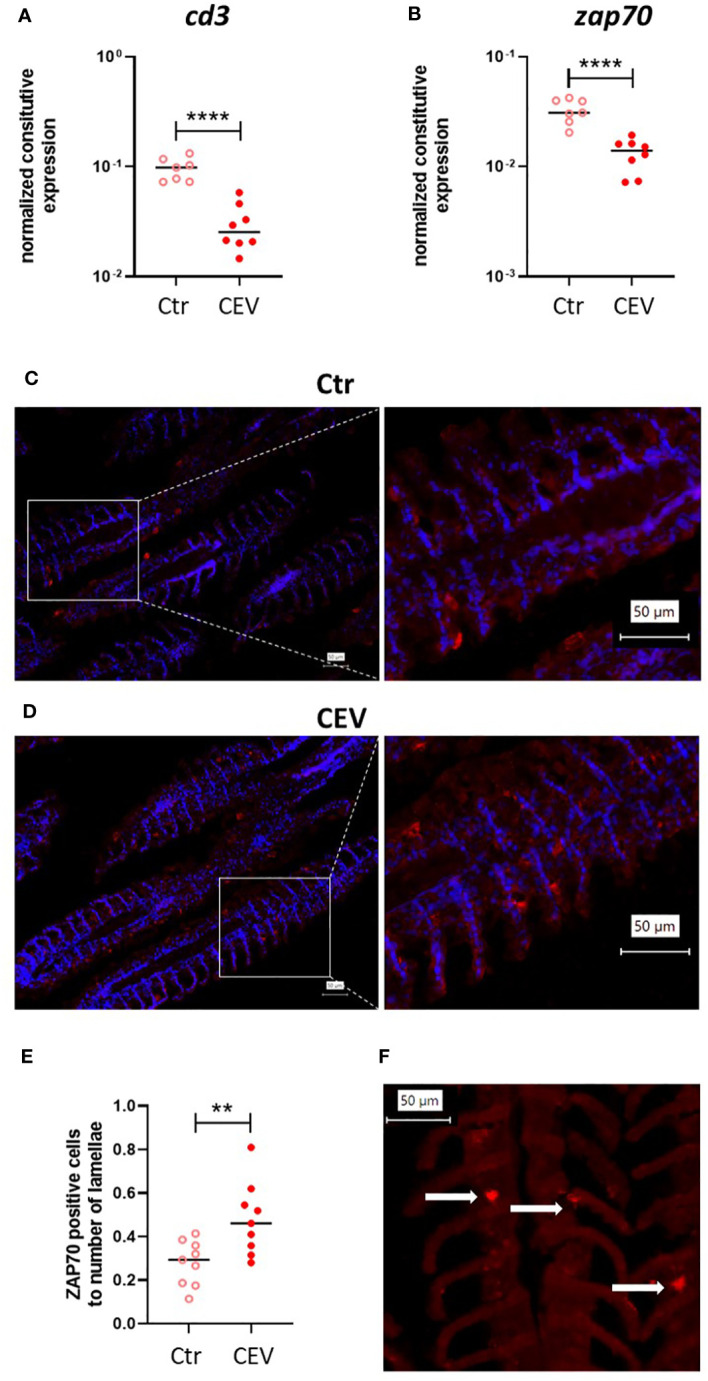
Expression of *cd3* **(A)** and *zap70* **(B)** in the gills of control (Ctr) and CEV-infected (CEV) koi kept at 18°C (6 days post exposure). Immunofluorescent staining of ZAP70-positive cells (in red) and nucleus (in blue) in the gills of control (Ctr) and CEV-infected (CEV) koi [**(C, D)**, respectively] and the ratio of the number of ZAP70-positive cells to the number of lamellae **(E)**. Only ZAP70-positive cells [**(F)** white arrows] associated with the secondary lamella were counted using digitally magnified images. The following procedure was used for counting ZAP70-positive cells: (i) counting the secondary lamellae, (ii) counting the positive cells on previously counted lamellae, (iii) counting the ratio of ZAP70-positive cells to the number of secondary lamellae. Asterisks indicate statistically significant differences between control and CEV-infected koi (**p ≤ 0.01; ****p ≤ 0.0001). Statistical analysis was performed using *t*-test. The data for gene expression are presented as n = 7–9 individual values with the mean indicated as a horizontal line. The data for ZAP70-positive cells are presented as three values from three different individuals with the mean indicated as a horizontal line.

Expression analysis of *cd3* and *zap70* genes combined with immunofluorescent staining using anti-ZAP70 antibodies (35) in the gills of CEV-infected and control koi, both kept at 18°C, was also performed to assess the effect of CEV infection on T cells in the gills (**Figures 9A–F**). Our results demonstrated a lower expression of *cd3* and *zap70* in the gills of CEV-infected koi compared to that in control uninfected koi (**Figures 9A, B**). However, immunofluorescent staining revealed a higher number of ZAP70-positive cells in the gills of CEV-infected koi (**Figures 9C–F**).

Our transcriptional analysis showed that CEV may affect immune responses depending on temperature, which correlates with viral load and KSD development. At both temperatures, we observed an upregulation of antiviral immune response genes in koi and salt-treated koi upon CEV infection, but it was only in infected koi at 18°C that we found clear CEV-induced downregulation of the expression of adaptive immune response genes. Whether this is accompanied by suppression of the immune response or is indicative of a redistribution of leukocytes following infection requires further investigation.

## 4 Discussion

Similar to other poxviruses that infect fish, such as salmon gill poxvirus (40) and *Plecoglossus altivelis* poxvirus (41), carp edema virus predominantly infects gills making it a very interesting model to study the effects of gill dysfunction on fish homeostasis. We hypothesized that disruption of the function of the gills during CEV infection of carp can cause physiological distress and impaired immunity in fish. The results of our previous studies suggest that CEV infection affects respiratory function of the gills (the level of blood oxygen was slightly reduced in CEV-infected koi), osmoregulation (sodium concentrations were markedly decreased in the blood plasma of CEV-infected koi), and metabolite removal (ammonia levels were significantly increased in the blood plasma of CEV-infected koi) (14). In the present study, we also observed significant changes in blood parameters at both 12°C and 18°C, e.g., decreased sodium and calcium concentrations, higher potassium concentration and pH, but also significant shifts in blood gas parameters. Lower sodium concentrations and an increase in free amino acids in the plasma of CEV-infected koi were also observed in previous studies (15). Changes in these blood parameters might not only be a result of impaired gill function but may also be related to a stress response.

Activation of the stress axis can be an important factor in the development of infection. It was demonstrated that carp strains with higher response to restraint stress were more susceptible to infection with the blood parasite *Trypanoplasma borreli* and the Gram-negative bacteria *Aeromonas hydrophila* (28). Using the restraint stress model, we compared the stress response between koi and Amur sazan. In both carp lines, restraint stress resulted in increased levels of the stress hormone cortisol and glucose in the blood plasma. It also induced similar pattern of expression of genes encoding proteins involved in the activation of the stress axis with the exception of the *mr* and *gr1* (encoding cortisol receptors). The expression of these genes was higher in NPO of stressed koi than in stressed Amur carp. However, many significant differences in stress response were observed between koi and Amur sazan during CEV infection. Our study demonstrated increased levels of cortisol and glucose in the blood plasma of CEV-infected fish at both temperatures, which correlated with increased viral load in the gills. Salt treatment of CEV-infected koi resulted in lower viral load in the gills and lower cortisol and glucose levels in the blood plasma compared to CEV-infected koi without salt treatment. In the resistant Amur sazan carp line, CEV infection also slightly increased the cortisol and glucose level in the blood plasma, although it was at a much lower level compared to that in the susceptible koi carp and only detectable in fish kept at 18°C. These observations are in accordance with previous studies reporting increased cortisol levels following infection of salmon with infectious pancreatic necrosis virus (IPNV) (42) and viral hemorrhagic septicemia virus (VHSV) (43). Moreover, several studies demonstrated that injection of fish with cortisol prior to infection resulted in higher susceptibility to pathogens (42, 44, 45). For example, after the experimental infection of salmon with SGPV, the disease progressed only if fish were pre-injected with hydrocortisone, but the increase in endogenous cortisol was also observed during SGPV infection (44). Interestingly, we found a higher basal level of cortisol in koi than in Amur sazan. This observation, together with the results of a previous study on SGPV infection in Atlantic salmon (44), may suggest that a higher basal level of cortisol in koi may be one of the reasons for their higher susceptibility to CEV infection. There is, therefore, a two-way relationship between cortisol and disease development.

In addition, cortisol also plays a role in osmoregulation in fish. As fish lack aldosterone, cortisol regulates Na^+^/K^+^-ATPase (NKA) activity in gills upon binding to GR and MR receptors. Cortisol induces the activation of NKA and also increases salinity tolerance (25). Interestingly, in our study, we observed a downregulation of *mr* expression in gills of CEV-infected koi subjected to salt treatment. However, restraint stress did not affect the expression of this gene in koi, which were not treated with salt.

The hypothesis of a CEV-induced stress response was also supported by the results of the expression analysis of genes involved in the activation of the HPI stress axis. Expression of these genes was upregulated especially in NPO of CEV-infected koi and to a lesser extent in salt-treated CEV-infected koi both kept at 18°C, whereas we did not observe many differences in the expression of stress axis-related genes in CEV-infected Amur sazan. Interestingly, upon CEV infection at 18°C, *gr1* expression was upregulated only in the NPO, while its expression was downregulated in other organs. In the NPO, the expression of g*r1* and *gr2* is part of a negative feedback loop that regulates the activity of the stress axis in response to cortisol release (46). However, the expression of these genes may not even be altered at all or may decrease after stress depending on the fish species and strain (28, 46). At 12°C, we did not observe altered expression of stress axis-related genes during CEV infection in the groups of fish studied, with a few exceptions. Taken together, our results clearly demonstrate that CEV infection induces the activation of the stress axis in fish resulting in increased levels of blood plasma cortisol. Activation of the stress axis correlated with the CEV viral load, which was higher at 18°C.

At both temperatures, CEV infection induced upregulation of the expression of most of the genes encoding proteins involved in innate immune response (oxysterol pathway, antiviral and inflammatory response), apoptosis, and cell stress. However, at 18°C, some of these genes, including *pkz*, *mpo*, *casp6*, *casp9*, and *fel*, were downregulated in CEV-infected koi. The protein kinase containing Z-DNA-binding domain (PKZ) (found in salmonids and cyprinids) is a paralog of the dsRNA-dependent protein kinase (PKR) (found in vertebrates) and activates the type I IFN-dependent antiviral immune response (47). Downregulation of *pkz* may be a possible mechanism by which CEV modulates the host response. A similar phenomenon was previously described for vaccinia virus, which encodes the E3L protein known to disrupt the PKR activity in mammals (48, 49). To date, the presence of the gene encoding E3L protein was not reported in piscine poxviruses, although this gene is present in most members of the *Chordopoxviridae* (poxviruses that infect vertebrates). *Mpo* is a neutrophil marker encoding for myeloperoxidase, an enzyme involved in innate immune response mechanisms (50). *Mpo* was also downregulated in SGPV-infected Atlantic salmon during clinical disease and later stage of infection (51). This shows that both viruses can affect the neutrophil response in the gills making it an interesting target for future investigations. However, high levels of granulocytosis were observed in the blood during CEV infection (14) demonstrating that gene expression alone does not always correspond to the cellular dynamics at the whole-body level. The *casp6* and *casp9* genes are involved in apoptosis. *Casp6* encodes executioner caspase 6, while *casp9* encodes initiator caspase 9. In contrast to SGPV infection, which leads to a pronounced upregulation of caspase genes and induction of apoptosis (51), CEV may have a different effect. In CEV-infected koi at 18°C downregulation of *casp6* and *casp9* expression and low levels of apoptotic cells in the gills were observed, which is in line with our previous results (5, 33). These data strongly suggest that apoptosis does not play a major role in KSD-related pathology.

Interestingly, in CEV-infected koi kept at 18°C, a clear downregulation of the expression of the genes encoding proteins involved in the adaptive immune response was observed. A previous study also showed a downregulation of the expression of *cd4*, *tcra2*, and *igm* in CEV-infected koi (14). Our present study confirmed this observation and showed that the expression of other adaptive immune genes, such as *tcra1*, *cd8a1*, *cd8b1*, and *cd8b2*, was also downregulated in the gills of CEV-infected koi kept at 18°C. Moreover, in the gills of CEV-infected koi kept at 18°C, downregulation of the expression of *cd3* (T-cell marker) and *zap70* (expressed on all T cells and NK cells) was observed, although immunofluorescent staining revealed a higher number of ZAP70-positive cells. Only the ZAP70-positive cells associated with the secondary lamella were counted, but there are other areas of high T-cell density in the gills [e.g., the interbranchial lymphoid tissue (ILT)] that were not included in our analysis. This could explain the discrepancy between ZAP70 protein detection and gene expression. Therefore, we could only speculate whether and how CEV modulates T-cell activity during infection. Mammalian poxviruses are commonly known for mechanisms that allow them to evade the host immune response (52); however, such mechanisms have not been well studied in the case of fish poxviruses. One of these mechanisms is the suppression of the host’s adaptive immune response, in particular, the T-cell response by the transmembrane proteins belonging to the B22 family of proteins found in several poxviruses (53). The B22 proteins have been well studied in mammalian poxviruses and found responsible for rendering T cells unresponsive to stimulation of T-cell receptor (TCR) in both MHC-dependent and -independent manner. This suppression of T-cell activity by B22 proteins is most likely mediated by cell–cell contact (53). Both CEV and SGPV possess, respectively, eight (54) and three (55) ortholog genes similar to *B22* of the Variola virus. In experimental trials, the expression of *B22* ortholog genes was demonstrated in the gills of SGPV-infected Atlantic salmon (56) and in the gills of CEV-infected koi (Adamek et al., unpublished data) already at 1 day post infection. Further research is needed to investigate in detail the effect of CEV infection on the adaptive immunity of carp. For this reason, FACS counting of T and B cells in the gills and other organs (e.g., spleen, kidney) and blood, as well as immunohistochemical analysis using pan-T-cell markers and anti-IgM, in combination with markers of apoptosis, should be performed to show changes in T- and/or B-cell abundance, distribution within the organs, and possibly their death.

The lower levels of T-cell marker expression may be related to the immunomodulatory effects of CEV genes, whose function has already been identified in other poxviruses. However, it is not only the virus that can influence the immune response. The pathophysiological stressors (osmotic disruption and ammonia intoxication) could also play a potent role in immunomodulation, as the fish treated with salt did not experience the downregulation of T-cell markers, as has been previously shown (14). Indeed, our present study demonstrates that the downregulation of the expression of the adaptive immune genes during CEV infection could also be associated with an increased stress response, which was at the highest level in clinically affected koi at 18°C. The stress response can be an important factor in the suppression of the immune response *via* its main signaling product, cortisol (57). Hydrocortisone injection in Atlantic salmon prior to SGPV infection resulted in delayed expression of antiviral gene *mx1* at the beginning of infection and decreased expression of genes encoding T-cell markers (*cd8α* and *cd4*) and cytotoxic immune effector genes (*ifnγ* and *gzma*) during the course of infection in the gills (56). In another study, slow release of cortisol implants resulted in impaired innate immune response and higher infection prevalence during infection of Atlantic salmon with infectious pancreatic necrosis virus (IPNV) (42).

In conclusion, our findings provide insight into the pathophysiology developing during the course of KSD. Changes in gill morphology, blood parameters, expression of stress-related and immune genes correlated with CEV viral load, which was greater at 18°C. Interestingly, CEV downregulated the expression of genes involved in the adaptive immune response, which is in line with previous studies following the factors affecting immunity: (i) poxviral capacity to modulate the host response, (ii) ammonia accumulation and osmotic disruption leading to lower leukocyte activity, and (iii) stress indicator (cortisol), which can also impair both innate and adaptive immunity. It is worth noting that the outcome of infection results from the interaction of several factors and may be additive. CEV infection at 18°C results in a higher viral load in the gills, which causes a disruption in the functions performed by this organ. This leads to stress and cortisol production that can lead to suppression of the immune response, ultimately favoring virus replication. In contrast, at 12°C, the virus replication is at a slightly lower level giving the fish immune system a chance to fight the infection.

## Data availability statement

The original contributions presented in the study are included in the article/**Supplementary Material**, further inquiries can be directed to the corresponding author/s.

## Ethics statement

The animal study was approved by 2nd Local Ethics Committee in Kraków, Poland (no. 355/2021). The study was conducted in accordance with the local legislation and institutional requirements.

## Author contributions

MZ: Conceptualization, Data curation, Formal analysis, Investigation, Methodology, Visualization, Writing – original draft, Writing – review & editing. AR: Data curation, Formal analysis, Investigation, Methodology, Writing – review & editing. FT: Investigation, Writing – review & editing. BK: Investigation, Writing – review & editing. VP: Resources, Writing – review & editing. DG: Resources, Writing – review & editing. MK: Resources, Writing – review & editing. MC: Conceptualization, Data curation, Formal analysis, Investigation, Methodology, Resources, Writing – review & editing. MA: Conceptualization, Data curation, Formal analysis, Funding acquisition, Investigation, Methodology, Project administration, Resources, Supervision, Visualization, Writing – original draft, Writing – review & editing. KR: Conceptualization, Data curation, Formal analysis, Funding acquisition, Investigation, Methodology, Project administration, Resources, Supervision, Visualization, Writing – original draft, Writing – review & editing.
