# Supplementary material for: Stressing out—carp edema virus induces stress and modulates immune response in common carp
Source: Front Immunol. 2024 Mar 21;15:1350197. doi: 10.3389/fimmu.2024.1350197 (PMC10991768; doi:10.3389/fimmu.2024.1350197)
Supplement: Supplementary file 1 [file Table_1.doc]

Supplementary Material

# Supplementary Tables

**Table S1.** Primers used for RT-qPCR and multiplex RT-qPCR. “R” indicates that primers were used for the gene expression analysis by RT-qPCR using Rotor Gene. “B” indicates that primers were used for the gene expression analysis by multiplex RT-qPCR using BioMark HD system (Standard Biotools).

| **Gene** | **Primer *forward* (5ʹ- 3ʹ)** | **Primer *reverse* (5ʹ- 3ʹ)** | **Reference** | **Use** |
| --- | --- | --- | --- | --- |
| *rps40* | CCGTGGGTGACATCGTTACA | TCAGGACATTGAACCTCACTGTCT | (32) | R |
| *ef1a* | ACAACCCCAAGGCTCTCAA | CCGCCAACTTTCTTCTCAAC | (14) | R |
| *crh* | CATCCGGCTCGGTAACAGAA | CCAACAGACGCTGCGTTAACT | (32) | R |
| *crhbp* | ACAATGATCTCAAGCGGTCCAT | CCACCCAGAAGCTCGACAAA | (32) | R |
| *crhr1* | CCCTGCTGATCGCCTTCAT | GCAGGATAAATGCTGTAATCAGGTT | (32) | R |
| *pomc* | TTGGCTCTGGCTGTTCTGTGT | TCATCTGTCAGATCAGACCTGCATA | (32) | R |
| *mc2r* | AGCATATTCCACGCGCTAGG | ACTGTAGCGGCCTCGAAGAA | (32) | R |
| *star* | GTGGAACCCCAATGTCAAAC | ACAGGTGGGTCCATTCTCAG | (32) | R |
| *cyp11b1* | CCCTGGAAGGTCAGTGTTGT | GGTGGGGTTTGGAGATAAGG | (32) | R |
| *il-1 β* | TAGGAGGCCAGTGGCTCTGT | CCTGAAGAAGAGGAGGCTGTCA | (32) | R |
| *gr1* | GACTTACCTGACTCCCTATCTGAC | GCTTCCACCATCTGCTGC | (32) | R |
| *gr2* | GGAGAACAACGGTGGGACTAAAT | GGCTGGTCCCGATTAGGAA | (32) | R |
| *mr* | TTCCCTGCAGAACTCAAAGGA | ACGGACGGTGACAGAAACG | (32) | R |
| *11βhsd1* | GCACTCAATGGTTTCTTTGGA | GCTCCAGCTTCGATAATGTG | (32) | R |
| *11βhsd2* | TACGCAAAAACAGCCAATGA | GTAGTATCGCACCTGGGGTTG | (32) | R |
| *mx2* | ATGACCCAGCAGAAGTGGAG | CAGGAACATTGGCAGAGATG |  | R |
| *tcra2* | CAGTGCGATATGCAGAACGAAGT | TTGACTGGATGATCCTTGCTGA | (14) | R |
| *cd4* | CGTGGACATCTGGCTTTGTG | TTTGGTTTTGCGTCGTCTGT | (14) | R |
| *cd8b1* | CGGCTCGGAAACTATCACCT | GAGTGGCGGACAGGTTTTCTC | (14) | R |
| *cd3* | TGAGTTGAGCGGTTTGACTG | TGGCAGCACAGAAGTAGATGA | (37) | R |
| *zap70* | GGAACAAGCCATCATTAGCC | GTCGTCTCTCACCCTCCTG | (35) | R |
| *ef1a* | ATTGGAGGTATTGGAACCGT | CAAAGGTCACAACCATACCTG | (38) | B |
| *actb* | GCATCAGGGAGTGATGGTT | GGCCTCATCTCCCACATAG | (38) | B |
| *rps11* | AAGGAGAAGCTCCCACGTTA | TGCCAGCAATAGCCTCTCT | (38) | B |
| *gapdh* | GCAAGCTGGTCATTGACG | GAGAGGGATCCAGCCAAC | (38) | B |
| *ch25h* | CCTGCCTGCTTCTCTTTGA | ACCTTATGGAAAGTGCGGTAC | (38) | B |
| *ch25h-like* | ACCACCTGGGCATTCATG | CGCCCAAGGGAAGTCATA | (38) | B |
| *cyp7b1* | CAGCAGTTTCTTTCACAACTACA | TGAATGTAATGTACCTTCCTGCTAT | (38) | B |
| *fdps* | GAGGGCAGAACTAGAGGCAT | CCGTTCAACACTTTCAGCA | (38) | B |
| *gpr183* | GGTGTGTTAGAAAACGTGGTTAAT | ATGGGGTCAGTTATGAAATCAA | (38) | B |
| *gig1* | TCAGAATCTCCCATACTATGAGG | AATAATGCGATCCTTGTTGCTGC | (38) | B |
| *ifna2* | TTAGATCAGATCATCAGCCTCAT | GTGTGGCCCAATACCAAA | (38) | B |
| *vig1* | CGGGACTATAAGGTGGCTTT | AAGACCTTCCAGCGCACT | (38) | B |
| *pkz* | ATCAGTTCCTGGCTAAGTGTGC | ATCTCCTCCAGCGTGCTAACAA | (38) | B |
| *irf7* | TCAAAACCACCAAGAGATCC | CAGACATGTAATTAGCTGCAGTG | (38) | B |
| *mx2* | AGGGAAGAGTTCTGTTCTCGA | GGCATCTTGTAACAATTCCACTA | (38) | B |
| *trim21* | CTTTGTGCAGTAAACGTGACTCT | TCTTCCCGAAGTGATGTGAGGT | (38) | B |
| *tcra1* | AACCACCAGCCTTTTACAAATTCA | AGTTTTCTGATCCACTAAAGCTGA | (38) | B |
| *tcra2* | TAAAAAAGAAGAACCGCCTGTCTA | CTGCTGTAATAACTTCGTTCTGC | (38) | B |
| *cd4* | ACCGAGAGTGAAGGTAGAAGAAA | TTCACGTCTTTCGCCCTTTGGA | (38) | B |
| *cd8a1* | CCAAAAATTGCTCCATTACC | CTGGGTTTGCCTTGTTTC | (38) | B |
| *cd8b1* | GGCTTCTATTCCTGCATGTTTA | AGGATTCACTCCAGGCATTAT | (38) | B |
| *cd8b2* | GGCTTCTATTCCTGCATGTTTA | AGGATTCACTCCAGGCATTAT | (38) | B |
| *il1-β1* | CCCAGATCAACTAGGCATGATG | TTCAACTTTTCCACAGCGATGAC | (38) | B |
| *il10* | ACTTGGAACCATTATTGAATGAA | GACGTTACATCCATAAGGACTATTG | (38) | B |
| *inos* | AGAAAGTTGCACTGCTGAAAA | GGTTCCCCATATTTACCTTTGT | (38) | B |
| *mpo* | TACCAGGCTATAATGCATGGCG | GCGATGCCTCCCAACCAAATG | (38) | B |
| *casp6* | ATGCCGTAGCCTTGTAGGG | ACGACAAGCCTGCAAGAT | (38) | B |
| *casp9* | TTCTGAAACTAAAGGGGTGAGA | CTGGCATCCATCTTATAACACTG | (38) | B |
| *tp53* | GTCGATGTCCCCATCATG | AGGAGGTGCCAATCCATC | (38) | B |
| *afap1* | CCTCTGTCCAGGCGATTCTGA | AGCCAGCCATGCCAAATACAGT | (38) | B |
| *iap/birc* | TTGGGAGTCTGGTGATGATC | CTTGCAACAGGTATTCACATCTT | (38) | B |
| *hsp70* | CGAGGCCATCAGCTGGCTAG | TGCTCCACGTGCCTGAGCTC | (38) | B |
| *serpinb1* | GGTGTGTCTGCTGCTCATATAA | CTGACACAATGCTGTAAAACG | (38) | B |
| *fel* | GCGGAGCAATGAAGGTTT | GTACAGTCTAAAGCCAGAGTGTGA | (38) | B |

**Table S2.** Constitutive gene expression of stress-involved genes in hypothalamic nucleus preopticus (NPO), pituitary gland (PIT), and head kidney (HK) of not stressed (Ctr) and stressed (Stress) koi and Amur sazan. The asterisk (*) indicates statistically significant differences between not stressed and stressed fish within the strain (* p ≤ 0.05; ** p ≤ 0.01; *** p ≤ 0.001) while different capital letters (e.g. A vs B) indicate statistically significant differences at p ≤ 0.05 between stressed koi and stressed Amur, as revealed by two-way ANOVA, with subsequent pairwise multiple comparisons using the Holm-Sidak test. The data are the mean±SD of n = 6 fish. NS- not studied.

| **genes** | **stress** | **NPO** | | **PIT** | | **HK** | |
| --- | --- | --- | --- | --- | --- | --- | --- |
|  |  | **Koi** | **Amur** | **Koi** | **Amur** | **Koi** | **Amur** |
| *crh* | Not stressed | 0.090± 0.027 | 0.076± 0.018 | NS | NS | NS | NS |
|  | Stressed | 0.085± 0.063 | 0.119± 0.070 | NS | NS | NS | NS |
| *crhbp* | Not stressed | 0.158± 0.038 | 0.170± 0.055 | 0.021± 0.029 | 0.034± 0.037 | NS | NS |
|  | Stressed | 0.214± 0.138 | 0.187± 0.068 | 0.020± 0.007 | 0.013± 0.012 | NS | NS |
| *crhr1* | Not stressed | 0.127± 0.070 | 0.107± 0.049 | 0.023± 0.012 | 0.025± 0.015 | NS | NS |
|  | Stressed | 0.139± 0.054 | 0.082± 0.028 | 0.029± 0.021 | 0.021± 0.013 | NS | NS |
| *pomc* | Not stressed | 0.006± 0.009 | 0.015± 0.022 | 4.807± 1.997 | 7.309± 5.462 | NS | NS |
|  | Stressed | 0.010± 0.022 | 0.009± 0.009 | 8.029± 4.346 | 17.679± 10.648 | NS | NS |
| *mr* | Not stressed | 0.102± 0.053 | 0.076± 0.038 | 0.030± 0.014 | 0.021± 0.014 | 0.004± 0.002 | 0.006± 0.002 |
|  | Stressed | **0.112± 0.024A** | **0.054± 0.030B** | 0.039± 0.019 | 0.024± 0.015 | 0.006± 0.004 | 0.006± 0.002 |
| *gr1* | Not stressed | 0.184± 0.156 | 0.147± 0.056 | 0.032± 0.012 | 0.049± 0.056 | 0.049± 0.020 | 0.046± 0.014 |
|  | Stressed | **0.127± 0.033A** | **0.070± 0.043*B** | 0.049± 0.047 | 0.021± 0.004 | 0.042± 0.025 | 0.036± 0.013 |
| *gr2* | Not stressed | 0.132± 0.051 | 0.098± 0.023 | 0.038± 0.013 | 0.045± 0.025 | 0.055± 0.020 | 0.062± 0.017 |
|  | Stressed | 0.141± 0.044 | 0.103± 0.050 | 0.049± 0.033 | 0.034± 0.017 | 0.051± 0.015 | 0.058± 0.008 |
| *il-1β* | Not stressed | 0.013± 0.009 | 0.005± 0.002 | 0.008± 0.008 | 0.004± 0.005 | 0.146± 0.058 | 0.106± 0.025 |
|  | Stressed | **0.069± 0.048***** | **0.050± 0.047**** | **0.031± 0.029*** | 0.008± 0.006 | 0.261± 0.115 | 0.142± 0.072 |
| *mc2r* | Not stressed | NS | NS | NS | NS | 0.001± 0.000 | 0.001± 0.001 |
|  | Stressed | NS | NS | NS | NS | 0.002± 0.001 | 0.003± 0.002 |
| *star* | Not stressed | NS | NS | NS | NS | 0.346± 0.199 | 0.282± 0.182 |
|  | Stressed | NS | NS | NS | NS | 0.437± 0.254 | 0.364± 0.319 |
| *cyp11b1* | Not stressed | NS | NS | NS | NS | 0.014± 0.008 | 0.024± 0.016 |
|  | Stressed | NS | NS | NS | NS | 0.009± 0.008 | **0.007± 0.006*** |
| *11βhsd2* | Not stressed | NS | NS | NS | NS | 0.073± 0.020 | 0.076± 0.030 |
|  | Stressed | NS | NS | NS | NS | **0.199± 0.053**** | **0.172± 0.077*** |
| *11βhsd1* | Not stressed | NS | NS | NS | NS | 0.004± 0.002 | 0.003± 0.001 |
|  | Stressed | NS | NS | NS | NS | 0.004± 0.001 | 0.002± 0.002 |

**Table S3.** Constitutive gene expression of stress-involved genes in hypothalamic nucleus preopticus (NPO), pituitary gland (PIT), and head kidney (HK) of three groups of carp: koi (Koi), salt-treated koi (Koi NaCl) and Amur sazan (Amur) kept at 12 °C. The asterisk (*) indicates statistically significant differences between not infected (Ctr) and CEV-infected (CEV) fish strain/within the group (* p ≤ 0.05; ** p ≤ 0.01; *** p ≤ 0.001). different small letters (e.g. a vs b) indicate statistically significant differences at p ≤ 0.05 between the groups of not infected fish. Different capital letters (e.g. A vs B) indicate statistically significant differences at p ≤ 0.05 between the groups of CEV-infected fish. Statistical analysis was performed using two-way ANOVA, with subsequent pairwise multiple comparisons using the Holm-Sidak test. The data are the mean±SD of n = 8 fish.

| **organ** | **genes** | **Koi** | | **Koi NaCl** | | **Amur** | |
| --- | --- | --- | --- | --- | --- | --- | --- |
|  |  | **Ctr** | **CEV** | **Ctr** | **CEV** | **Ctr** | **CEV** |
| NPO | *crh* | 0.015± 0.005 | 0.029±  0.019 | 0.030± 0.018 | 0.037±  0.024 | 0.031± 0.021 | 0.020±  0.014 |
|  | *crhbp* | 0.061± 0.035 | 0.049±  0.031 | 0.078± 0.060 | 0.048±  0.019 | 0.072± 0.048 | 0.048±  0.019 |
|  | *crhr1* | 0.078± 0.058 | 0.112±  0.074 | 0,09±  0.085 | 0,094±  0,062 | 0.05±  0.036 | 0.033±  0.03 |
|  | *pomc* | 0.003± 0.005 | 0.013±  0.016 | 0.007± 0.011 | 0.006±  0.007 | 0.008± 0.011 | 0.007±  0.009 |
|  | *mr* | 0.128± 0.063 | 0.123±  0.060 | 0.100± 0.060 | 0.107±  0.055 | 0.062± 0.030 | 0.105±  0.104 |
|  | *gr1* | 0.105± 0.098 | **0.136±**  **0.089A** | 0.098± 0.081 | **0.136±**  **0.101A** | 0.083± 0.058 | **0.049± 0.036B** |
|  | *gr2* | 0.024± 0.011 | **0.034±**  **0.015A** | 0.024± 0.013 | **0.033±**  **0.014A** | 0.016± 0.006 | **0.013± 0.007B** |
|  | *il-1β* | 0.014± 0.012 | **0.032±**  **0.043A** | 0.010± 0.007 | **0.014±**  **0.006AB** | 0.005± 0.003 | **0.019± 0.045B** |
| PIT | *crhbp* | 0.007± 0.007 | 0.015±  0.013 | 0.004± 0.004 | 0.030±  0.063 | 0.002± 0.002 | 0.005±  0.008 |
|  | *crhr1* | 0.001± 0.001 | 0.001±  0.001 | 0.001± 0.001 | 0.094±  0.261 | 0.001± 0.001 | 0.003±  0.005 |
|  | *pomc* | 15.225± 9.400 | 29.917±  18.209 | 15.612± 10.569 | 16.997±  9.566 | 19.569± 10.867 | 23.594± 10.754 |
|  | *mr* | 0.025± 0.009 | 0.032±  0.026 | 0.037± 0.030 | 0.028±  0.016 | 0.040± 0.031 | 0.034±  0.018 |
|  | *gr1* | 0.017± 0.003 | 0.015±  0.012 | 0.022± 0.007 | **0.013±**  **0.010*↓** | 0.019± 0.016 | 0.015±  0.006 |
|  | *gr2* | 0.026± 0.004 | 0.046±  0.016 | 0.041± 0.020 | 0.210±  0.501 | 0.039± 0.031 | 0.026±  0.010 |
|  | *il-1β* | 0.002± 0.001 | 0.013±  0.016 | 0.004± 0.006 | 0.003±  0.002 | 0.001± 0.001 | 0.011±  0.028 |
| HK | *mr* | 0.0007± 0.0002 | **0.0004± 0.0002*A↓** | 0.0007± 0.0003 | **0.0005± 0.0002A** | 0.0010± 0.0003 | **0.0027± 0.0035*B↑** |
|  | *gr1* | **0.006± 0.001a** | **0.004± 0.002*A↓** | **0.006± 0.001a** | **0.005±**  **0.001A** | **0.011± 0.009b** | **0.009± 0.002B** |
|  | *gr2* | **0.004± 0.001a** | **0.004±**  **0.001A** | **0.004± 0.001a** | **0.004±**  **0.001A** | **0.008± 0.005b** | **0.006± 0.001B** |
|  | *il-1β* | **0.037± 0.010a** | **0.061±**  **0.048A** | **0.021± 0.009ab** | **0.057± 0.017**A↑** | **0.018± 0.007B** | **0.009± 0.007**B↓** |
|  | *mc2r* | 0.0001± 0.0001 | 0.0001±  0.0002 | 0.0001± 0.0001 | 0.0001±  0.0001 | 0.0003± 0.0002 | 0.0001± 0.0001 |
|  | *star* | 0.013± 0.012 | 0.032±  0.025 | 0.040± 0.033 | 0.016±  0.011 | 0.038± 0.027 | 0.018±  0.014 |
|  | *cyp11b1* | 0.0004± 0.0004 | 0.0005±  0.0004 | 0.0007± 0.0008 | 0.0003±  0.0002 | 0.0009± 0.0006 | 0.0016± 0.0034 |
| GILLS | *mr* | **0.004±**  **0.001a** | **0.002±**  **0.001***A↓** | **0.006±**  **0.002ab** | **0.002±**  **0.001***A↓** | **0.012±**  **0.013b** | **0.006±**  **0.001B** |
|  | *gr1* | 0.016±  0.003 | 0.013±  0.005 | 0.018±  0.005 | 0.013±  0.003 | 0.020±  0.010 | 0.018±  0.008 |
|  | *gr2* | 0.008±  0.005 | 0.006±  0.003 | 0.009±  0.003 | 0.006±  0.002 | 0.007±  0.004 | 0.010±  0.003 |
|  | *il-1β* | 0.013±  0.011 | **0.105±**  **0.178**A↑** | 0.006±  0.002 | **0.036±**  **0.027**A↑** | 0.016±  0.011 | **0.009±**  **0.009B** |
|  | *11βhsd1* | 0.003±  0.001 | 0.002±  0.002 | 0.004±  0.002 | 0.003±  0.002 | 0.003±  0.002 | 0.003±  0.002 |
|  | *11βhsd2* | 0.154± 0.112 | 0.198±  0.131 | 0,200± 0,085 | 0.123±  0.032 | 0,212± 0,103 | 0.342±  0.118 |

**Table S4.** Constitutive gene expression of stress-involved genes in hypothalamic nucleus preopticus (NPO), pituitary gland (PIT), and head kidney (HK) of three groups of carp: koi (Koi), salt-treated koi (Koi NaCl) and Amur sazan (Amur) kept at 18 °C. The asterisk (*) indicates statistically significant differences between not infected (Ctr) and CEV-infected (CEV) fish within the strain/group (* p ≤ 0.05; ** p ≤ 0.01; *** p ≤ 0.001). Different small letters (e.g. a vs b) indicate statistically significant differences at p ≤ 0.05 between the groups of not infected fish. Different capital letters (e.g. A vs B) indicate statistically significant differences at p ≤ 0.05 between the groups of CEV-infected fish. Statistical analysis was performed using two-way ANOVA, with subsequent pairwise multiple comparisons using the Holm-Sidak test. The data are the mean±SD of n = 8 fish.

| **Organ** | **genes** | **Koi** | | **Koi NaCl** | | **Amur** | |
| --- | --- | --- | --- | --- | --- | --- | --- |
|  |  | **Ctr** | **CEV** | **Ctr** | **CEV** | **Ctr** | **CEV** |
| NPO | *crh* | **0.104± 0.028a** | **0.201± 0.068***↑** | **0.126± 0.016ab** | **0.203±**  **0.029**↑** | **0.151± 0.032b** | **0.211± 0.076*↑** |
|  | *crhbp* | 0.097± 0.032 | **0.190± 0.055***↑** | 0.109± 0.038 | **0.176±**  **0.060*↑** | 0.086± 0.024 | **0.143± 0.065*↑** |
|  | *crhr1* | 0.046± 0.009 | **0.107± 0.025***A↑** | 0.053± 0.018 | **0.090± 0.014***A↑** | 0.044± 0.010 | **0.054± 0.015B** |
|  | *pomc* | 0.010± 0.005 | **0.047± 0.015***A↑** | 0.022± 0.012 | **0.047±**  **0.018A** | 0.011± 0.001 | **0.017± 0.011B** |
|  | *mr* | 0.045± 0.011 | **0.071± 0.017***A↑** | 0.041± 0.006 | **0.084± 0.018***A↑** | 0.030± 0.014 | **0.038± 0.014B** |
|  | *gr1* | 0.094± 0.023 | **0.133±**  **0.034*↑** | 0.099± 0.023 | **0.161±**  **0.026**↑** | 0.095± 0.028 | **0.135± 0.049*↑** |
|  | *gr2* | 0.052± 0.009 | **0.109± 0.025***A↑** | 0.066± 0.011 | **0.094± 0.027*AB↑** | 0.061± 0.013 | **0.071± 0.024B** |
|  | *il-1β* | **0.010± 0.007ab** | **0.099± 0.064***A↑** | **0.018± 0.012a** | **0.025±**  **0.013B** | **0.006± 0.004b** | **0.007± 0.004C** |
| PIT | *crhbp* | 0.007± 0.005 | **0.021± 0.009*A↑** | 0.014± 0.022 | **0.017±**  **0.017A** | 0.003± 0.002 | **0.003± 0.002B** |
|  | *crhr1* | **0.034± 0.016a** | **0.014±**  **0.006A** | **0.044± 0.019a** | **0.036±**  **0.020B** | **0.070± 0.014b** | **0.076± 0.026C** |
|  | *pomc* | 14.867± 5.976 | 10.112±  6.423 | 6.699± 2.615 | 16.284±  5.711 | 20.721± 9.467 | 13.615± 4.788 |
|  | *mr* | 0.056± 0.013 | **0.021± 0.005**A↓** | 0.058± 0.019 | **0.049±**  **0.019B** | 0.042± 0.026 | **0.046± 0.022B** |
|  | *gr1* | 0.180± 0.064 | **0.041± 0.017***A↓** | 0.188± 0.067 | **0.118±**  **0.044*B↓** | 0.179± 0.034 | **0.180± 0.048C** |
|  | *gr2* | 0.072± 0.030 | 0.094±  0.022 | 0.095± 0.043 | 0.090±  0.031 | 0.098± 0.020 | 0.092± 0.031 |
|  | *il-1β* | 0.003± 0.003 | **0.112± 0.129***A↑** | 0.006± 0.003 | **0.010±**  **0.010B** | 0.005± 0.005 | **0.003± 0.002C** |
| HK | *mr* | 0.004± 0.002 | **0.001± 0.001***A↓** | 0.005± 0.004 | **0.003±**  **0.002B** | 0.003± 0.002 | **0.003± 0.001B** |
|  | *gr1* | 0.059± 0.025 | **0.030±**  **0.014*** | 0.063± 0.036 | 0.040±  0.023 | 0.052± 0.015 | 0.049± 0.018 |
|  | *gr2* | 0.040± 0.016 | 0.028±  0.008 | 0.043± 0.027 | 0.035±  0.016 | 0.035± 0.009 | 0.043± 0.017 |
|  | *il-1β* | 0.075± 0.024 | **0.440± 0.494***A↑** | 0.088± 0.051 | **0.068±**  **0.010B** | 0.060± 0.016 | **0.059± 0.011B** |
|  | *mc2r* | 0.002± 0.001 | 0.001±  0.001 | 0.003± 0.001 | 0.005±  0.011 | 0.001± 0.001 | 0.003± 0.002 |
|  | *star* | 0.431± 0.202 | **0.508±**  **0.326A** | 0.435± 0.260 | **0.157±**  **0.078*B↓** | 0.272± 0.182 | **0.496± 0.304A** |
|  | *cyp11b1* | 0.015± 0.009 | **0.014±**  **0.021A** | 0.031± 0.021 | **0.031±**  **0.072*B↓** | 0.015± 0.010 | **0.035± 0.030A** |
| GILLS | *mr* | 0.005±  0.004 | 0.002±  0.001 | 0.007±  0.004 | **0.003±**  **0.002*↓** | 0.009±  0.008 | 0.004±  0.002 |
|  | *gr1* | 0.050±  0.015 | **0.029±**  **0.007*↓** | 0.053±  0.015 | **0.030±**  **0.015*↓** | 0.055±  0.020 | 0.047±  0.017 |
|  | *gr2* | 0.040±  0.021 | **0.021±**  **0.004*A↓** | 0.045±  0.012 | **0.027±**  **0.011**AB↓** | 0.042±  0.011 | **0.038±**  **0.010B** |
|  | *il-1β* | 0,008± 0,002 | **0,557**± **0,348***A↑** | 0,013± 0,009 | **0,103**± **0,115**B↑** | 0,008± 0,005 | **0,021**± **0,021C** |
|  | *11βhsd2* | **0.023±**  **0.009a** | **0.015±**  **0.007A** | **0.013±**  **0.004a** | **0.024±**  **0.016A** | **0.021±**  **0.011b** | **0.012±**  **0.012B** |
|  | *11βhsd1* | 0.032±  0.016 | 0.025±  0.009 | 0.019±  0.006 | 0.026±  0.027 | 0.076±  0.039 | 0.057±  0.026 |

**Table S5**. Relative transcripts abundance of immune related genes in gills of not infected (Ctr) and CEV-infected (CEV) fish from three groups of carp: koi (Koi), salt-treated koi (Koi NaCl) and Amur sazan (Amur) kept at 12 °C. The asterisk (*) indicates statistically significant differences between not infected and infected within the strain/group (* p ≤ 0.05; ** p ≤ 0.01; *** p ≤ 0.001). Different small letters (e.g. a vs b) indicate statistically significant differences at p ≤ 0.05 between the groups of not infected fish. Different capital letters (e.g. A vs B) indicate statistically significant differences at p ≤ 0.05 between the groups of CEV-infected fish. Statistical analysis was performed using two-way ANOVA, with subsequent pairwise multiple comparisons using the Holm-Sidak test. The data are the mean±SD of n = 8 fish.

| **Genes** | **Koi** | | **Koi NaCl** | | **Amur** | |
| --- | --- | --- | --- | --- | --- | --- |
|  | **Ctr** | **CEV** | **Ctr** | **CEV** | **Ctr** | **CEV** |
| *ch25h* | 1.1E+06 ± 3.6E+05 | **2.4E+07 ± 2.6E+07***A** | 2.6E+06± 2.3E+06 | **3.2E+07 ± 1.6E+07***A** | 3.1E+06 ± 3.6E+06 | **2.1E+06 ± 1.2E+06B** |
| *ch25h-like* | 5.2E+05 ± 2.4E+05 | 1.0E+07 ± 1.0E+07 | 1.2E+06 ± 1.0E+06 | 1.6E+07 ± 9.1E+06 | 1.6E+06 ± 1.6E+06 | 1.0E+06 ± 5.0E+05 |
| *cyp7b1* | 8.2E+05 ± 2.3E+05 | **2.0E+06 ± 6.2E+05***A** | 1.3E+06 ± 5.0E+05 | **3.0E+06 ± 2.1E+06***A** | 1.1E+06 ± 5.0E+05 | **1.0E+06 ± 3.1E+05B** |
| *fdps* | **8.6E+06 ± 2.4E+06a** | **2.5E+07 ± 9.1E+06***A** | **1.1E+07 ± 2.5E+06ab** | **3.0E+07 ± 4.5E+06***A** | **1.6E+07 ± 4.1E+06b** | **1.4E+07 ± 4.5E+06B** |
| *gig1* | 9.8E+05 ± 4.4E+05 | **8.3E+07 ± 5.1E+07***A** | 1.5E+06 ± 5.1E+05 | **1.1E+08 ± 3.9E+07***A** | 7.1E+05 ± 2.9E+05 | **2.3E+06 ± 2.8E+06*B** |
| *gpr183* | 8.4E+04 ± 3.9E+04 | **1.9E+05 ± 5.8E+04***A** | 8.9E+04 ± 3.2E+04 | **2.2E+05 ± 7.4E+04***A** | 1.2E+05 ± 4.7E+04 | **9.0E+04 ± 3.7E+04B** |
| *ifna2* | 5.8E+05 ± 3.1E+05 | **1.2E+07 ± 1.8E+07***A** | 6.2E+05 ± 3.5E+05 | **1.5E+07 ± 8.2E+06***B** | 1.1E+06 ± 7.4E+05 | **1.7E+06 ± 9.5E+05C** |
| *vig1* | 1.8E+06 ± 8.5E+05 | **3.4E+08 ± 2.6E+08***A** | 2.5E+06 ± 1.2E+06 | **4.1E+08 ± 1.4E+08***A** | 3.0E+06 ± 2.1E+06 | **1.3E+07 ± 2.0E+07B** |
| *pkz* | 6.2E+05 ± 2.1E+05 | **1.1E+06 ± 4.3E+05***A** | 6.2E+05 ± 1.0E+05 | **1.2E+06 ± 3.6E+05***A** | 8.5E+05 ± 2.4E+05 | **6.3E+05 ± 1.7E+05B** |
| *irf7* | 2.6E+06 ± 1.0E+06 | **3.6E+07 ± 2.4E+07***A** | 3.8E+06 ± 1.1E+06 | **4.1E+07 ± 8.7E+06***A** | 3.7E+06 ± 1.4E+06 | **9.2E+06 ± 6.7E+06**B** |
| *mx2* | 1.5E+06 ± 7.9E+05 | **8.1E+07 ± 5.0E+07***A** | 2.0E+06 ± 1.1E+06 | **1.0E+08 ± 1.5E+07***A** | 1.9E+06 ± 1.9E+06 | **9.0E+06 ± 1.1E+07**B** |
| *trim21* | 1.0E+06 ± 3.9E+05 | 4.0E+08 ± 3.3E+08 | 8.5E+05 ± 9.3E+05 | 4.0E+08 ± 1.1E+08 | 4.1E+05 ± 6.4E+05 | 5.5E+06 ± 1.1E+07 |
| *tcra1* | 2.0E+06 ± 5.8E+05 | **3.2E+06 ± 9.8E+05*** | 2.3E+06 ± 4.8E+05 | 3.4E+06 ± 1.4E+06 | 2.4E+06 ± 1.1E+06 | 2.5E+06 ± 1.3E+06 |
| *tcra2* | **1.8E+06 ± 7.2E+05a** | **1.9E+06 ± 6.8E+05A** | **1.9E+06 ± 6.7E+05a** | **2.6E+06 ± 1.1E+06A** | **9.2E+05 ± 2.3E+05b** | **1.2E+06 ± 7.6E+05B** |
| *cd4* | 6.3E+05 ± 2.9E+05 | 9.9E+05 ± 3.9E+05 | 6.6E+05 ± 2.4E+05 | **1.3E+06 ± 9.1E+05*** | 1.1E+06 ± 6.7E+05 | 1.2E+06 ± 7.1E+05 |
| *cd8a1* | 7.7E+06 ± 4.2E+06 | **1.1E+07 ± 3.2E+06A** | 6.2E+06 ± 1.5E+06 | **1.5E+07 ± 9.3E+06**A** | 6.1E+06 ± 3.3E+06 | **6.4E+06 ± 2.9E+06B** |
| *cd8b1* | **1.2E+06 ± 5.2E+05a** | **2.0E+06 ± 5.3E+05*** | **1.8E+06 ± 9.3E+05a** | **3.0E+06 ± 1.5E+06*** | **3.5E+06 ± 9.9E+05b** | 3.3E+06 ± 1.4E+06 |
| *cd8b2* | **1.2E+06 ± 6.0E+05a** | **2.2E+06 ± 5.5E+05**A** | **2.0E+06 ± 1.1E+06b** | **3.5E+06 ± 1.7E+06**AB** | **3.8E+06 ± 1.1E+06c** | **3.9E+06 ± 1.6E+06B** |
| *igm* | 4.2E+06 ± 2.5E+06 | **8.8E+06 ± 4.0E+06*AB** | 6.1E+06 ± 3.2E+06 | **1.4E+07 ± 5.4E+06*A** | 4.3E+06 ± 1.8E+06 | **3.4E+06 ± 1.8E+06B** |
| *il1b1* | 3.1E+05 ± 1.3E+05 | **8.8E+06 ± 2.1E+07***A** | 3.3E+05 ± 1.0E+05 | **1.7E+06 ± 1.7E+06**A** | 5.2E+05 ± 2.2E+05 | **3.2E+05 ± 1.1E+05B** |
| *il10* | **1.1E+05 ± 9.3E+04a** | **1.0E+06 ± 9.2E+05***A** | **9.3E+04 ± 5.0E+04a** | **7.9E+05 ± 4.2E+05***A** | **4.3E+04 ± 4.4E+04b** | **8.5E+04 ± 6.4E+04B** |
| *inos* | 6.2E+04 ± 3.4E+04 | 1.7E+06 ± 1.9E+06 | 2.2E+05 ± 1.7E+05 | 1.7E+06 ± 7.8E+05 | 1.9E+05 ± 2.1E+05 | 1.9E+05 ± 1.7E+05 |
| *mpo* | 1.9E+06 ± 1.7E+06 | **6.8E+06 ± 4.7E+06*** | 2.2E+07 ± 1.4E+06 | 4.2E+06 ± 3.1E+06 | 3.4E+06 ± 1.7E+06 | 6.0E+06 ± 4.0E+06 |
| *casp6* | 2.0E+07 ± 4.2E+06 | **3.6E+07 ± 1.8E+07*A** | 2.2E+07 ± 5.6E+06 | **3.4E+07 ± 1.3E+07*A** | 2.0E+07 ± 5.9E+06 | **1.6E+07 ± 4.7E+06B** |
| *casp9* | **9.6E+03 ± 2.1E+03a** | **2.0E+04 ± 9.3E+03**** | **1.5E+04 ± 4.8E+03a** | 1.6E+04 ± 5.6E+03 | **2.5E+04 ± 9.5E+03b** | **1.7E+04 ± 9.8E+03*** |
| *tp53* | 9.2E+06 ± 2.9E+06 | **1.5E+07 ± 3.3E+06***AB** | 1.0E+07 ± 2.9E+06 | **1.7E+07 ± 4.0E+06**A** | 1.1E+07 ± 2.4E+06 | **1.1E+07 ± 3.4E+06B** |
| *afap1* | **6.0E+06 ± 1.8E+06a** | **1.3E+07 ± 2.7E+06***** | **8.9E+06 ± 1.5E+06b** | **1.3E+07 ± 2.4E+06**** | **9.5E+06 ± 2.4E+06b** | 1.0E+07 ± 3.1E+06 |
| *iap/birc* | **7.0E+06 ± 2.7E+06a** | **3.1E+07 ± 1.4E+07***A** | **1.1E+07 ± 3.8E+06b** | **2.4E+07 ± 5.1E+06***A** | **7.6E+06 ± 1.2E+06ab** | **7.2E+06 ± 1.8E+06B** |
| *hsp70* | 2.6E+05 ± 2.6E+05 | **3.1E+06 ± 3.0E+06***A** | 2.7E+05 ± 2.1E+05 | **1.7E+06 ± 1.1E+06***A** | 3.1E+05 ± 2.8E+05 | **7.7E+05 ± 6.2E+05B** |
| *serpinb1* | **5.3E+06 ± 1.1E+06a** | **8.0E+06 ± 2.5E+06*** | **6.1E+06 ± 1.4E+06a** | 7.4E+06 ± 9.8E+05 | **9.5E+06 ± 3.3E+06b** | 8.8E+06 ± 3.2E+06 |
| *fel* | **9.2E+06 ± 7.5E+06a** | **2.3E+07 ± 1.7E+07**AB** | **2.4E+07 ± 2.0E+07b** | **1.1E+07 ± 6.6E+06A** | **3.9E+07 ± 1.4E+07b** | **3.5E+07 ± 2.0E+07B** |

**Table S6.** Relative transcripts abundance of immune related genes in gills of not infected (Ctr) and CEV-infected (CEV) fish from three groups of carp: koi (Koi), salt-treated koi (Koi NaCl) and Amur sazan (Amur) kept at 18 °C. The asterisk (*) indicates statistically significant differences between not infected and infected fish within the strain/group (* p ≤ 0.05; ** p ≤ 0.01; *** p ≤ 0.001). Different small letters (e.g. a vs b) indicate statistically significant differences at p ≤ 0.05 between the groups of not infected fish. Different capital letters (e.g. A vs B) indicate statistically significant differences at p ≤ 0.05 between the groups of CEV-infected fish. Statistical analysis was performed using two-way ANOVA, with subsequent pairwise multiple comparisons using the Holm-Sidak test. The data are the mean±SD of n = 8 fish.

| **Genes** | **Koi** | | **Koi NaCl** | | **Amur** | |
| --- | --- | --- | --- | --- | --- | --- |
|  | **Ctr** | **CEV** | **Ctr** | **CEV** | **Ctr** | **CEV** |
| *ch25h* | **4.6E+06 ± 2.1E+06a** | **5.2E+07 ± 2.3E+07***A** | **5.6E+06 ± 2.3E+06a** | **1.2E+07 ± 5.5E+06**B** | **7.7E+05 ± 4.5E+05b** | **8.6E+05 ± 3.9E+05C** |
| *ch25h-like* | **2.3E+06 ± 9.3E+05a** | **2.3E+07 ± 1.0E+07***A** | **2.4E+06 ± 1.1E+06a** | **5.7E+06 ± 3.1E+06**B** | **6.8E+05 ± 2.3E+05b** | **8.2E+05 ± 3.5E+05C** |
| *cyp7b1* | 1.7E+06 ± 7.9E+05 | 2.2E+06 ± 9.8E+05 | 2.1E+06 ± 1.0E+06 | 1.8E+06 ± 7.8E+05 | 1.3E+06 ± 7.2E+05 | 1.7E+06 ± 6.3E+05 |
| *fdps* | 1.7E+07 ± 2.4E+06 | **7.5E+07 ± 5.3E+07***A** | 1.5E+07 ± 6.2E+06 | **3.0E+07 ± 1.7E+07**B** | 1.1E+07 ± 2.4E+06 | **1.1E+07 ± 2.9E+06C** |
| *gig1* | **8.9E+06 ± 9.7E+06a** | **1.6E+08 ± 4.7E+07***A** | **4.2E+06 ± 2.2E+06a** | **1.1E+08 ± 1.1E+08***A** | **1.1E+06 ± 8.6E+05b** | **7.3E+06 ± 4.3E+06***B** |
| *gpr183* | 3.5E+05 ± 6.3E+04 | 3.5E+05 ± 1.2E+05 | 3.0E+05 ± 7.5E+04 | 3.4E+05 ± 6.6E+04 | 2.7E+05 ± 9.0E+04 | 2.1E+05 ± 6.3E+04 |
| *ifna2* | 2.6E+06 ± 1.2E+06 | **1.9E+07 ± 1.1E+07***A** | 1.8E+06 ± 7.5E+05 | **5.0E+06 ± 3.9E+06**B** | 2.4E+06 ± 1.3E+06 | **3.1E+06 ± 1.2E+06B** |
| *vig1* | 1.9E+07 ± 1.7E+07 | **3.1E+08 ± 9.9E+07***A** | 1.1E+07 ± 3.6E+06 | **1.8E+08 ± 1.9E+08***B** | 7.2E+06 ± 5.9E+06 | **1.7E+07 ± 1.3E+07C** |
| *pkz* | **1.1E+06 ± 2.3E+05a** | **8.2E+05 ± 1.5E+05**A** | **9.7E+05 ± 2.1E+05a** | **1.3E+06 ± 1.5E+05*B** | **6.8E+05 ± 1.1E+05b** | **6.4E+05 ± 1.2E+05C** |
| *irf7* | 1.1E+07 ± 5.1E+06 | **3.5E+07 ± 9.2E+06***A** | 8.3E+06 ± 2.3E+06 | **3.1E+07 ± 1.4E+07***A** | 6.4E+06 ± 2.9E+06 | **1.1E+07 ± 5.7E+06*B** |
| *mx2* | **1.7E+07 ± 1.1E+07a** | **9.4E+07 ± 8.7E+06***A** | **9.1E+06 ± 3.1E+06ab** | **6.3E+07 ± 4.3E+07***B** | **5.5E+06 ± 3.8E+06b** | **1.3E+07 ± 7.6E+06*C** |
| *trim21* | **1.8E+07 ± 1.9E+07a** | **4.4E+08 ± 1.8E+08***A** | **6.3E+06 ± 4.5E+06a** | **1.6E+08 ± 1.7E+08***B** | **1.8E+06 ± 1.7E+06b** | **6.1E+06 ± 3.8E+06*C** |
| *tcra1* | 1.1E+07 ± 3.0E+06 | **2.6E+06 ± 1.7E+06***A** | 9.4E+06 ± 3.8E+06 | **5.2E+06 ± 2.1E+06B** | 8.2E+06 ± 2.3E+06 | **7.5E+06 ± 1.6E+06B** |
| *tcra2* | **5.7E+06 ± 1.2E+06a** | **1.1E+06 ± 7.4E+05***A** | **4.1E+06 ± 1.0E+06ab** | **2.6E+06 ± 7.4E+05B** | **3.0E+06 ± 9.5E+05b** | **2.6E+06 ± 4.5E+05B** |
| *cd4* | 1.2E+06 ± 2.3E+05 | **5.2E+05 ± 2.5E+05***A** | 8.8E+05 ± 1.6E+05 | **7.2E+05 ± 3.0E+05AB** | 1.1E+06 ± 4.2E+05 | **1.1E+06 ± 4.5E+05B** |
| *cd8a1* | 4.1E+07 ± 2.5E+07 | **1.1E+07 ± 6.7E+06**A** | 2.9E+07 ± 2.7E+07 | **3.6E+07 ± 2.4E+07B** | 2.5E+07 ± 8.6E+06 | **2.5E+07 ± 6.9E+06B** |
| *cd8b1* | 6.9E+06 ± 4.8E+06 | **3.2E+06 ± 1.2E+06**A** | 5.8E+06 ± 2.3E+06 | **7.4E+06 ± 6.8E+06AB** | 7.5E+06 ± 1.8E+06 | **7.0E+06 ± 2.2E+06B** |
| *cd8b2* | 8.9E+06 ± 6.0E+06 | **3.8E+06 ± 1.5E+06**A** | 7.3E+06 ± 3.0E+06 | **8.5E+06 ± 7.2E+06AB** | 9.2E+06 ± 2.2E+06 | **8.8E+06 ± 2.8E+06B** |
| *igm* | 2.7E+07 ± 1.7E+07 | **1.2E+07 ± 3.6E+06AB** | 2.4E+07 ± 1.1E+07 | **3.2E+07 ± 2.1E+07A** | 1.2E+07 ± 6.9E+06 | **1.2E+07 ± 1.4E+07B** |
| *il1b1* | 5.9E+05 ± 1.2E+05 | **2.1E+07 ± 3.2E+07***A** | 5.1E+05 ± 2.7E+05 | **4.1E+05 ± 1.1E+05B** | 2.7E+05 ± 8.3E+04 | **5.2E+05 ± 1.7E+05B** |
| *il10* | **3.1E+05 ± 2.1E+05a** | **4.5E+05 ± 2.4E+05A** | **1.4E+05 ± 8.9E+04ab** | **2.6E+05 ± 2.1E+05AB** | **6.8E+04 ± 1.6E+04b** | **1.1E+05 ± 5.7E+04B** |
| *inos* | 1.0E+05 ± 7.1E+04 | 2.7E+05 ± 2.3E+05 | 1.5E+05 ± 1.2E+05 | 2.1E+05 ± 2.2E+05 | 8.4E+04 ± 5.1E+04 | 2.1E+05 ± 3.8E+05 |
| *mpo* | 6.7E+06± 3.7E+06 1.5 | **7.9E+05± 6.3E+05***A** | 6.8E+06± 2.5E+06 | **4.0E+06 ± 2.1E+06B** | 4.5E+06± 1.8E+06 | **7.7E+06 ± 3.0E+06C** |
| *casp6* | **5.9E+07 ± 1.5E+07a** | **3.4E+07 ± 8.9E+06***A** | **6.6E+07 ± 3.3E+07a** | **5.7E+0 7± 1.2E+07B** | **2.8E+07± 5.8E+06b** | **3.6E+07 ± 8.4E+06A** |
| *casp9* | 5.8E+04 ± 1.5E+04 | **2.9E+04 ± 1.2E+04***A** | 4.6E+04 ± 1.3E+04 | **4.6E+04 ± 1.3E+04B** | 7.1E+04 ± 2.5E+04 | **5.1E+04 ± 1.5E+04B** |
| *tp53* | **1.8E+07 ± 1.7E+06a** | **1.7E+07 ± 2.0E+06AB** | **1.7E+07 ± 3.7E+06ab** | **1.8E+07 ± 3.6E+06A** | **1.4E+07 ± 1.7E+06b** | **1.4E+07 ± 2.4E+06B** |
| *afap1* | 1.2E+07 ± 3.5E+06 | 1.0E+07 ± 1.3E+06 | 9.6E+06 ± 3.2E+06 | 9.8E+06 ± 1.5E+06 | 9.5E+06 ± 1.0E+06 | 1.2E+07 ± 2.2E+06 |
| *iap/birc* | **2.2E+07 ± 8.2E+06a** | **3.4E+07 ± 1.3E+07*A** | **2.6E+07 ± 1.4E+07a** | **2.8E+07 ± 8.6E+06A** | **9.7E+06 ± 1.5E+06b** | **1.0E+07 ± 2.0E+06B** |
| *hsp70* | 3.4E+05 ± 2.6E+05 | **2.2E+06 ± 1.0E+06***A** | 3.4E+05 ± 3.3E+05 | **1.4E+06 ± 1.5E+06B** | 2.4E+05 ± 1.3E+05 | **3.1E+05 ± 1.4E+05B** |
| *serpinb1* | 1.0E+07 ± 3.1E+06 | 8.1E+06 ± 1.5E+06 | 9.1E+06 ± 4.0E+06 | 8.7E+06 ± 2.7E+06 | 8.6E+06 ± 1.8E+06 | 8.8E+06 ± 1.6E+06 |
| *fel* | 6.6E+07 ± 6.2E+07 | **1.2E+07 ± 1.1E+07***A** | 6.4E+07 ± 4.2E+07 | **2.5E+07 ± 2.0E+07 *B** | 4.7E+07 ± 1.0E+07 | **7.1E+07 ± 5.0E+07 C** |

**
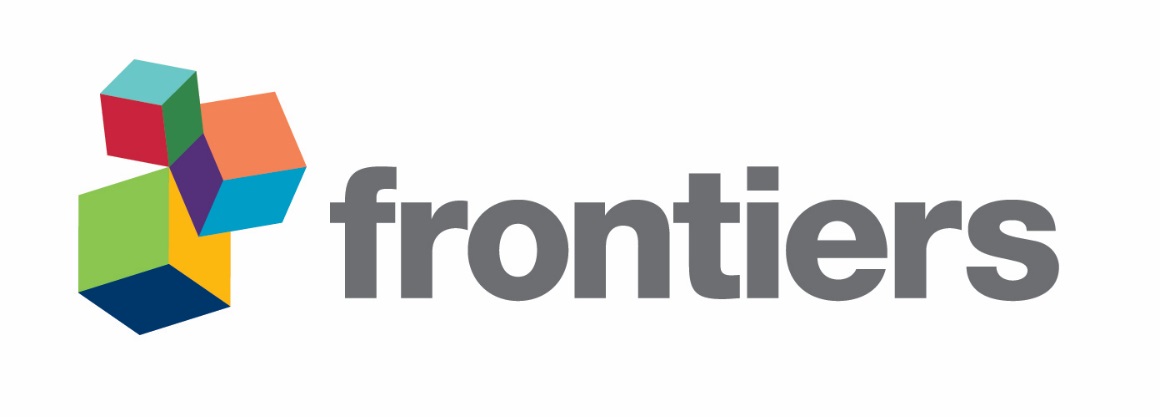
**
